# Diastereoselective Synthesis and Biological Evaluation of Spiro[chromane-2,4′-pyrimidin]-2′(3′*H*)-ones as Novel Antimicrobial and Antioxidant Agents

**DOI:** 10.3390/molecules30142954

**Published:** 2025-07-14

**Authors:** Alena S. Karandeeva, Natalia A. Bogdanova, Mariya V. Kabanova, Sergey I. Filimonov, Zhanna V. Chirkova, Anna A. Romanycheva, Valeria A. Panova, Anton A. Shetnev, Nurila A. Togyzbayeva, Saken A. Kanzhar, Nurbol O. Appazov, Kyrill Yu. Suponitsky

**Affiliations:** 1 Institute of Chemistry and Chemical Technology, Yaroslavl State Technical University, Moskovskii Prosp. 88, 150023 Yaroslavl, Russiabogdanovana.20@yandex.ru (N.A.B.); mariya_vk02@mail.ru (M.V.K.); filimonovsi@ystu.ru (S.I.F.); chirkovazhv@ystu.ru (Z.V.C.); 2 Pharmaceutical Technology Transfer Centre, Yaroslavl State Pedagogical University Named After K.D. Ushinsky, Respublikanskaya Str. 108, 150000 Yaroslavl, Russia; kai-ren@yandex.ru (A.A.R.); lero4ka_aria@mail.ru (V.A.P.); 3 Moscow Center for Advanced Studies, Kulakova Str. 20, 123592 Moscow, Russia; 4 Laboratory of Engineering Profile, Korkyt Ata Kyzylorda University, Aiteke Bi Str., 29A, 120014 Kyzylorda, Kazakhstan; sakenkanzhar@gmail.com (S.A.K.); nurasar.82@korkyt.kz (N.O.A.); 5 A. N. Nesmeyanov Institute of Organoelement Compounds, Russian Academy of Sciences, Build. 1, Vavilova Str. 28, 119991 Moscow, Russia; kirshik@yahoo.com

**Keywords:** heterocycles, spirochromane-pyrimidines, resorcinol, 2-methylresorcinol, pyrogallol, acid catalysis, diastereoselective synthesis, antimicrobial activity, antioxidant activity, pharmacokinetics

## Abstract

This study reports an improved diastereoselective synthesis of substituted spiro[chromane-2,4′-pyrimidin]-2′(3′*H*)-ones via the acid-catalyzed condensation of 6-styryl-4-aryldihydropyrimidin-2-ones with resorcinol, 2-methylresorcinol, and pyrogallol. The optimized method allows for the isolation of diastereomerically pure products, with stereoselectivity controlled by varying acid catalysts (e.g., methanesulfonic acid vs. toluenesulfonic acid) and solvent conditions. The synthesized compounds were evaluated for antimicrobial and antioxidant activities. Notably, the (2S*,4R*,6′R*)-diastereomers exhibited significant antibacterial activity against both Gram-positive and Gram-negative bacterial strains with minimal inhibition concentration down to 2 µg/mL, while derivatives containing vicinal bisphenol moieties demonstrated potent antioxidant activity, with IC_50_ values (12.5 µg/mL) comparable to ascorbic acid. Pharmacokinetic analysis of selected hit compounds revealed favorable drug-like properties, including high gastrointestinal absorption and blood-brain barrier permeability. These findings highlight the potential of spirochromane-pyrimidine hybrids as promising candidates for further development in the treatment of infectious diseases and oxidative stress-related pathologies.

## 1. Introduction

The search for new chemotypes to overcome the global threat of bacterial multidrug resistance is becoming increasingly important year by year. In recent years, a number of key structural motifs for scaffold-based drug design of new antimicrobial agents have been identified. Some of them were spirocompounds [1], chromanes with phenolic groups [2], substituted cyclic ureas and pyrimidines [3]. The mechanisms underlying the bioactivities of these scaffolds’ derivatives are multifaceted and often involve their interactions with specific molecular targets. For instance, the antimicrobial activity of phenol derivatives is attributed to their disruption of microbial membranes, which leads to cell death [4].

Spirochromans demonstrate diverse biological activities [1,5], particularly when incorporating nitrogen-containing heterocycles [6]. This type of heterocycle exhibits acetyl-CoA-carboxylase (ACC) inhibition properties, used as an anti-inflammatory, antihypertensive, antiplasmodial, antimalarial agent [7], as an antioxidant [8], has antitumor activity [9], and analgesic properties [10].

The effect of spirocycles on biological activity is often attributed to the escape from flatland to provide a less planar molecule with improved potency [11]. The spirocyclic quaternary carbon positioned at the center of a bioactive molecule offers conformational rigidity, which in turn reduces the penalty for conformational entropy [12]. Owing to their inherent three-dimensionality and structural novelty, spiro scaffolds have been increasingly used in drug discovery [5,11]. More than what the spirocyclic motif is frequently found in natural compounds [13]. In addition, the spirocyclic compound with a well-defined three-dimensional arrangement has a greater possibility of being the drug candidate appearing in clinical trials [14].

The phenolic groups in the molecule play a major role in imparting biological activity. The bioactivities of phenol derivatives encompass a broad spectrum of therapeutic applications, ranging from antimicrobial, antioxidant, and anti-inflammatory effects to anticancer, neuroprotective, and cardioprotective properties [15]. These diverse activities stem from their ability to modulate cellular processes, such as cell growth, apoptosis, immune response, and oxidative stress. Moreover, the structural versatility of phenol derivatives allows for the development of specific compounds targeting particular biological pathways or diseases [16]. Polyphenolic compounds encompass many substances that are produced through the secondary metabolism of plants, fungi, and bacteria, in addition to being produced by chemical synthesis [17,18]. Studies indicate that these compounds are associated with several pharmacological activities, including antibacterial activity [19]. The presence of phenolic groups in the chromane moiety increases the probability of antibacterial activity [16]. Plant polyphenols with pyrogallol groups have a more significant activity than polyphenols [16,20] with catechol or resorcinol groups; as such, pyrogallol group presence is an indicator for potent antibacterial activity [21,22].

**Figure 1:**
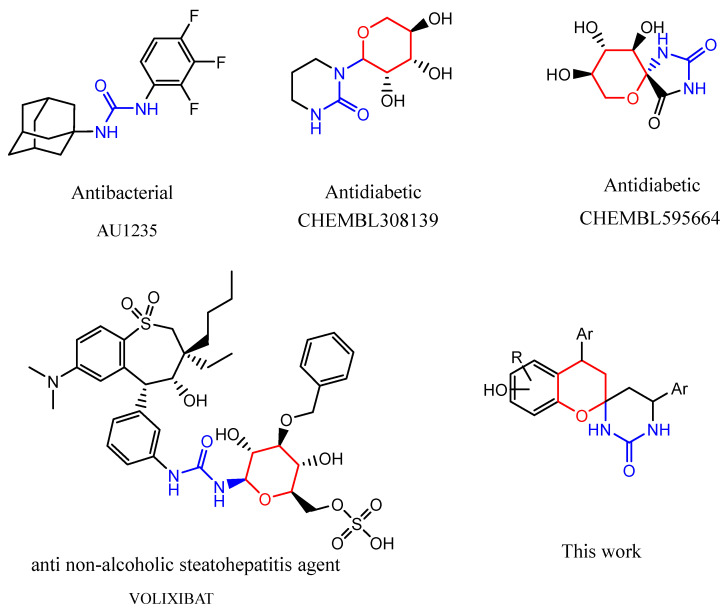
Examples of pharmaceutical important chromanes or 2H-pyran core and ureas.

Additionally, compounds containing a pyrimidine ring exhibit a wide range of biological activities, including antibacterial activity [3]. It was also found that chromeno[3,2-*i*]quinazolines and spirochromanes with a urea fragment similar in structure exhibit antibacterial properties [23,24,25]. The combination of phenolic pyrimidine and chroman moieties coupled with the spirocyclic type of fusion may provide an interesting biological profile for target molecules (Figure 1).

For example, antidiabetic agents CHEMBL308139 [26] and CHEMBL595664 [27] from the chemical database of bioactive molecules of the European Molecular Biology Laboratory (EMBL), based on a spirocyclic combination of five- and six-membered ureas and tetrahydro-2H-pyran core. The clinical candidate volixibat for the treatment of nonalcoholic steatohepatitis contains a tetrahydro-2H-pyran core bearing an aryl-substituted urea moiety [28].

In this paper, we propose for consideration a new spirocyclicspiro[chromane-2,4′-pyrimidin]-2′(3′*H*)-one-containing scaffold, which can be positioned as a modification of the successful anti-tuberculosis drug candidate AU1235 [29,30].

Syntheses of spirochromanes from styrylpyrimidines were first described by G. Zigeuner [31,32,33]. Subsequent methodological developments for spirochromane synthesis were advanced by our research group [34,35]. Various synthetic approaches have been reported for spirochromanes, including routes using phenol derivatives as starting materials, cycloaddition [36], chiral catalysts [37], and conventional acid catalysis [38,39]. Recently, novel methods for styrylpyrimidine synthesis have been developed, involving heating of classical Biginelli reaction products with aromatic aldehydes at 80–100 °C. This yields 4,6-diaryl-3,4-dihydropyrimidin-2(1H)-ones in satisfactory yields with broad flexibility for aryl substituent variation [40,41,42].

## 2. Results and Discussion

### 2.1. Chemistry

**Scheme 1:**
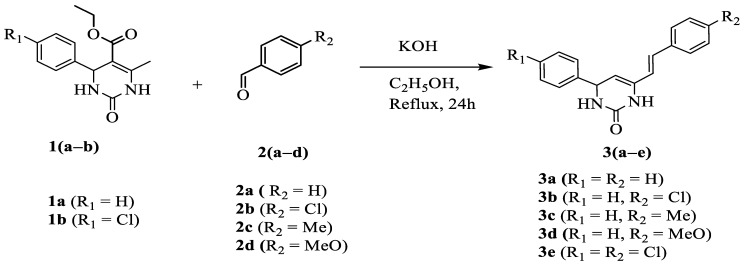
Synthesis of starting 6-stiryl-4-aryldihydropyrimidin-2-ones.

Starting 6-stiryl-4-aryldihydropyrimidin-2-ones **3a**–**e** were prepared via strong base-activated condensation of Biginelli compounds **1a**–**b** with aldehydes **2a**–**d** [40] (Scheme 1).

Frequently, reactions of styryldihydropyrimidines with resorcinols result in the formation of spirochromanepyrimidines, usually in the form of two diastereomers [34,35]. The synthesis of spiro[chroman-2,4′-pyrimidine]-2′(3′*H*)-ones, which are formed as two (2R*,4R*,6′R*)/(2S*,4R*,6′R*)-diastereoisomers in the condensation of substituted 6-steryl-4-aryldihydropyrimidin-2-ones and resorcinol under acid catalysis with *p*-TsOH, was reported earlier [43]. It was found that under these conditions, the formation of (2R*,4R*,6′R*)-diastereoisomer (upfield) prevails, for which the location of the nearest nitrogen atom in the same plane with the chromane cycle (equatorially) is determinant according to the RSA data. The second (downfield) diastereoisomer could not be isolated for electron-donating substituents of styrylpyrimidines, as well as spirochromanes with the fragment pyrogallol.

We were able to solve this problem with the replacement of *p*-TsOH acid with CH_3_SO_3_H. However, pyrogallol-based spirochromans were usually isolated as a mixture of diastereomers in a ratio of approximately 1:1.

**Scheme 2:**
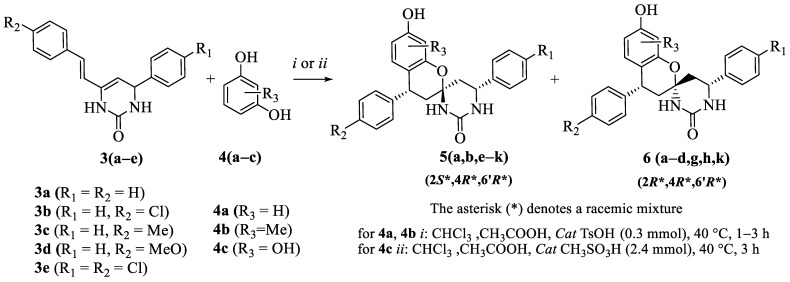
Synthesis of target spiro[chromane-2,4′-pyrimidin]-2′(3′*H*)-ones **5** and **6**.

In continuation of the work, using the developed synthesis methods, the target series of diastereomerically pure spirocyclic products **5** and **6** (Scheme 2) was obtained. The isolated yields of compounds **5** and **6** are summarized in Table 1.

In view of the importance of developing methods for obtaining diastereomerically pure products, we carried out a deep optimization of the method for synthesizing target spiro[chromane-2,4′-pyrimidin]-2′(3′*H*)-ones using the example of the interaction of chlorine-substituted styrylpyrimidine with pyrogallol. The results of the experiments for finding the optimal reaction conditions are summarized in Table 2.

The study (Table 2) showed that when using only CH_3_SO_3_OH (*Entry* 7) as an acid catalyst, diastereomer **5** is mainly formed. When using a mixture of acids CH_3_SO_3_H (1.2 mmol), TsOH*H_2_O (1.2 mmol), diastereomer **6** is formed exclusively (*Entry* 14). In both cases, the yield does not exceed 42 and 32%, respectively. The optimal ratio of CH_3_SO_3_OH/AcOH for obtaining the maximum yield was also determined (*Entry* 3). It should be noted that when using pure TsOH*H_2_O as a catalyst, the reaction with pyrogallols does not occur.

Thus, using CH_3_SO_3_OH/AcOH, it was possible to obtain spirochromanes with an extended range of resorcinol and substituents 2 in the ratio (2R*,4R*,6′R*)/(2S*,4R*,6′R*)-diastereoisomers 1:1 with satisfactory yield more than 70% (Scheme 2). This allowed us to shorten the reaction time and to obtain the product as a 1:1 mixture of diastereomers, not only with resorcinol **4** but also with pyrogallol. The products obtained from pyrogallol **5**, **6** showed similar physicochemical properties as in the case of spirochromanes on resorcinol. The (2S*,4R*,6′R*)-diastereoisomer is poorly soluble in ethyl acetate and is easily isolated from the mixture. This made it possible to separate the mixtures by fractional crystallization from ethyl acetate into individual (2R*,4R*,6′R*) or (2S*,4R*,6′R*) diastereomers with a diastereomeric purity of more than 95% for most of the obtained compounds.

It should be noted that styrylpyrimidines with donor substituents turned out to be the most sensitive to this catalytic system, since large concentrations of CH_3_SO_3_OH led to resinification of the reaction mixture. Therefore, for the synthesis of chromanes based on pyrogallol, the optimal synthesis conditions were selected, see Table 1. Based on the studies performed, the concentration of **2.4** mmol CH_3_SO_3_OH was found to be the most suitable one.

The structure of the obtained **5**, **6** spirochromans was confirmed by combined NMR spectroscopy and mass spectrometry. However, compounds **6** (upfield) are characterized by more upfield chemical shifts of 4-H and 6′-H protons in the region 4.0–4.5 ppm in NMR ^1^H spectra and C-spiro signal in the range of 84.1–84.9 ppm ^13^C NMR spectra. For diastereomers **5**, these downfield signals correspond in the region 4.60–5.30 m.d. in ^1^H NMR and C-spiro signal in the region 82.10–82.20 ppm in ^13^C NMR spectra. For the (2S*,4R*,6′R*)-diastereomer of compound **5h**, the structure was unambiguously confirmed by RSA data, and it was shown that for this diastereomer, the axial position of the nitrogen atom relative to the chromane cycle is characteristic.

### 2.2. X-Ray Studies

The structure of **5h** was established by single-crystal X-ray diffraction. Single-crystal samples were grown from DMF solution by slow evaporation in the form of very thin plates with extremely low reflection ability. It crystallizes in the form of a solvate with 1.5 molecules of DMF and half of a water molecule in the centrosymmetric space group *C*2/*c*. Molecule **5h** contains three chiral carbon atoms (C7, C9, C11, according to X-ray numbering). Relative configuration is *SRR*(*RSS*).

**Figure 2:**
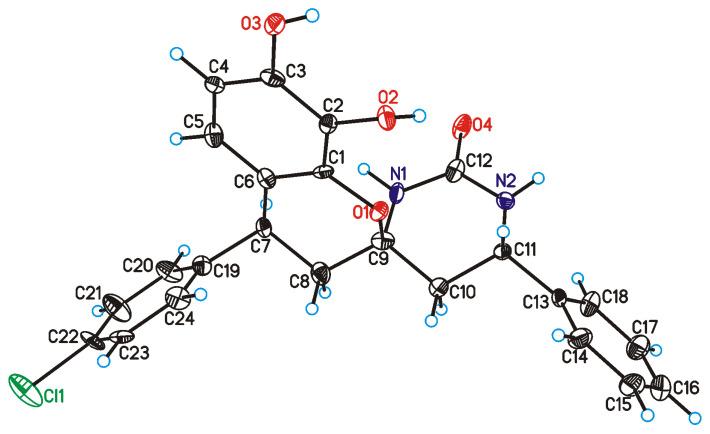
General view of compound **5h** showing numbering scheme. Molecular visualizations use the CPK atomic color convention (C: grey, N: blue, O: red, Cl: green). Thermal ellipsoids are given at 50% probability level.

Single-crystal X-ray diffraction analysis reveals that the compound **5h** crystallizes in the centrosymmetric space group *C*2/*c*, confirming its racemic nature” (Figure 2).

### 2.3. Biological Studies

The previously obtained and newly developed 16 diastereomerically pure spiro[chromane-2,4′-pyrimidin]-2′(3′*H*)-ones were selected to study their potential for use as antimicrobial agents. The antimicrobial, hemolytic, and antibiofilm activity of the obtained derivatives **5** and **6** was studied. In addition, the antimicrobial activity of the starting styrylpyrimidines **3a**–**e** was investigated.

#### 2.3.1. Minimal Inhibition Concentration (MIC)

In accordance with the recommendations [44], the antimicrobial effect of the synthesized compounds **3a**–**e**, **5a**–**k** and **6a**–**k** on 8 strains of sensitive bacteria and 1 strain of fungi was studied using the disk diffusion method and the double serial dilution method. Styrylpyrimidines **3a**–**e** did not show antibacterial activity up to a concentration of 256 µg/mL. The obtained results for compounds **5**–**6** are presented in Table 3.

Notably, only the (2S*,4R*,6′R*)-diastereomers (**5**), derived from resorcinol and 2-methylresorcinol, exhibited significant antimicrobial activity. The spatial spiro factor plays a decisive role in the activity of these compounds. For spirochromans obtained from pyrogallol, both diastereoisomers exhibit high activity, but a slight advantage also remains for (2S*,4R*,6′R*)-diastereomers. The best activity indices were demonstrated by spirochromans with R_1_ = H, R_2_ = Cl. Interesting selectivity of antimicrobial activity towards Gram “−” bacteria was demonstrated by both diastereomers of spirochromans with R_1_ = Cl, R_2_ = Cl substituents on pyrogallol. Probably, aryl substituents play a major role in pyrogallol, which can affect both the configuration of the molecule and its interaction with the membrane of microbes.

The observed variations in antimicrobial activity may arise from structural differences in bacterial cell walls. In gram-negative bacteria, the presence of hydrophilic pores, a smaller cell wall thickness may make it a less pronounced barrier for compounds, which may determine a more significant antimicrobial effect in **5i**–**5k**. This is also indicated by a more significant value of Log P: >5.

The obtained data indicate the presence of significant antimicrobial activity and potential for further pharmaceutical development of **5f**; **6g**, **5h**; **6h**; **5b** as antimicrobial drug candidates.

#### 2.3.2. Hemolytic Activity

To evaluate the potential undesired toxic effects on eukaryotic cells, the hemolytic activity of the hit compounds **5b**, **5f**, **5h**, and **6h** was assessed on human red blood cells.

**Figure 3:**
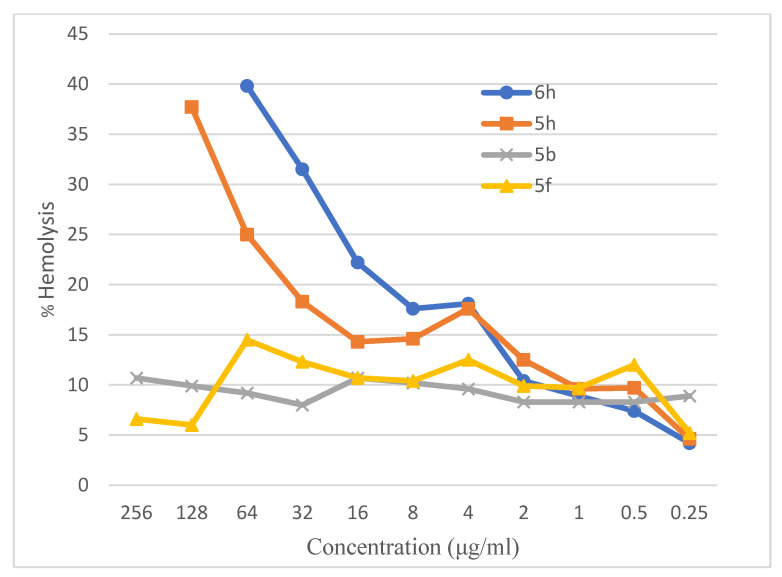
Hemolytic activity of hit compounds **5b**, **5f**, **5h**, and **6h** against red human blood cells.

Figure 3 shows the results of tests to determine the hemolytic activity of hit compounds **5b**, **5f**, **5h**, and **6h**.

The studied compounds in concentrations up to 32 μg/mL have virtually no hemolytic effect on human erythrocytes. However, in high concentrations of 256-128-64 μg/mL, the studied substances cause erythrocyte agglutination. In most cases, the presence of hemagglutinating activity in a potential antibacterial agent is considered an undesirable property, since it may indicate a non-specific interaction of molecules with host cells and probably increases the risk of toxicity. To optimize safety, it is necessary to minimize or eliminate such properties while maintaining antibacterial activity. For clarity, comparative data on hemolytic activity at a concentration of 32 are presented in Table 4.

All the procedures were carried out thrice. Data are mean ± standard deviation (*n* = 3). Triton was utilized as a standard. P—Statistical significance.

Hit-compounds **5f**; **5h**; **6h**; **5b** have virtually no hemolytic effect on erythrocytes at concentrations up to 32 μg/mL; At concentrations of 256-128-64 μg/mL, compounds **5f**; **5h**; **6h**; **5b** cause agglutination of erythrocytes, which may indicate a non-specific interaction of these compounds with the cells of the host organism.

#### 2.3.3. Antibiofilm Activity

In the 24-h preformed biofilm alone, *Staphylococcus aureus* and *Escherichia coli* were insensitive to the hit-compounds **5f**, **5h**, **6h**, and **5b**. However, the compounds may be useful in preventing biofilm formation. This means that the compounds prevent biofilm formation on non-biofilm-containing surface layers.

#### 2.3.4. Pharmacokinetics Analysis

The two hit compounds **5b**, **5f**, exhibit high gastrointestinal (GI) absorption and Blood-Brain Barrier (BBB) permeability, indicating potential for bioavailability and central nervous system (CNS) penetration. Compounds **5h**, **6h** showed no Blood-Brain Barrier (BBB) permeability and one PAIN alert, indicating potential binding with cathechol amines. These predictions highlight favorable drug-like properties with potential challenges related to promiscuity and metabolic inhibition. Molecules adhere to Lipinski’s rule of five with two violations, assessed with molecular weight >300 (Their molecular weights (MW) range from 421 to 455.) and LogP > 3.5 (LogP 3.53…4.42) and do not dimiss drug-likeness.

Regarding metabolic interactions, all inhibit CYP2C19 and CYP2C9, with variability in inhibition of CYP2D6 and CYP3A4 presented in Table 5. These predictions highlight favorable drug-like properties with potential challenges related to promiscuity and metabolic inhibition. The StopTox toxicity parameters were used to assess the safety of compounds based on various exposure routes, including oral and dermal toxicity, acute inhalation, and effects on eye and skin irritation. All tested compounds showed one negative prediction associated with potential eye irritation and corrosion. Based on other toxicological parameters, no negative predictions were calculated.

Bioavailability radar and ADME analysis of compounds (**5b**, **5f**, **5h**, **6h**) using the server are in the Supplementary Information.

### 2.4. Antioxidant Assay

Due to the characteristic structural features of the synthesized compounds (polyphenol groups), it was expected that these compounds would exhibit antioxidant activity. DPPH, a free radical cleaning method, was used in this study to measure antioxidant capacity [45]. Ascorbic acid is the most commonly used standard due to its potent scavenging activity. We tested 4 initial hit compounds using the DPPH method adapted for use on 96-unit plates. Table 6 shows the DPPH radical scavenging activity results of the tested compounds compared to ascorbic acid.

It was shown that two compounds containing a vicinal bisphenol fragment, **5h**, **6h,** were found to have antioxidant activity comparable to ascorbic acid IC_50_ = 12.5 μg/mL, while monophenol derivatives **5b**, **5f** in the concentration range of 400–6.5 μg/mL showed no activity.

## 3. Experimental

### 3.1. Materials and Methods

NMR spectra were recorded on a Bruker DRX-400 (Bruker-Biospin, Karlsruhe, Germany) and a Bruker DRX-500 (Bruker-Biospin, Karlsruhe, Germany) instrument in DMSO-*d6* at 30 °C. The chemical shifts are given in the δ scale relative to the residual solvent signals δH 2.50 (1H) and δC 39.5 (13C) and recalculated to SiMe_4_ (ZIOC RAS, Moscow, Russia). IR spectra were collected in the reflected light mode in the 700–4000 cm^−1^ range using a PerkinElmer Spectrum Two FR-IR spectrometer (PerkinElmer Inc., Shelton, CT, USA). Mass spectrometry was carried out on a FINNIGAN MAT INCOS 50 (Finnigan MAT, Hemel Hempstead, UK) GC/MS/DS system (ionization energy of 70 eV, ion source temperature of 100–220 °C) (ZIOC RAS, Moscow, Russia). Elemental analysis was carried out on a Perkin Elmer 2400 CHNS/O (PerkinElmer Inc.) elemental analyzer (Laboratory of Microanalysis, INEOS RAS, Moscow, Russia). Melting points were measured on a Buchi M-560 (BUCHI Labortechnik AG, Flawil, Switzerland) apparatus.

### 3.2. Synthesis and Characterization of 6-Stiryl-4-Aryldihydropyrimidin-2-Ones

Compounds **3a**–**d** were obtained according to the Shutalev method [41]; the physicochemical characteristics of these structures are described in the work [40,41,42]. Compounds **5a**,**b**,**e,** and **6a**–**d** were obtained according to method [43].

4-phenyl-6-[(*E*)-2-phenylethenyl]-3,4-dihydropyrimidin-2(1*H*)-one (**3a**) [42]. Beige powder; 87% yield; m.p. 198–201 °C. ^1^H NMR (400 MHz, DMSO-d_6_) δ 8.40 (s, 1H), 7.18–7.47 (m, 11H), 7.09 (d, J = 16.61 Hz, 1H), 6.62 (d, J = 16.85 Hz, 1H), 5.09 (d, J = 14.41 Hz, 2H).

6-[(*E*)-2-(4-chlorophenyl)ethenyl]-4-phenyl-3,4-dihydropyrimidin-2(1*H*)-one (**3b**) [42]. Pale yellow powder; 87% yield; m.p. 207–209 °C. ^1^H NMR (400 MHz, DMSO-d_6_) δ 8.41 (s, 1H), 7.22–7.48 (m, 10H), 7.07 (d, J = 16.61 Hz, 1H), 6.65 (d, J = 16.55 Hz, 1H), 5.10 (d, J = 9.10 Hz, 2H).

6-[(*E*)-2-(4-methylphenyl)ethenyl]-4-phenyl-3,4-dihydropyrimidin-2(1*H*)-one (**3c**) [40]. Beige powder; 93% yield; m.p. 214–218 °C. ^1^H NMR (400 MHz, DMSO-d_6_) δ 8.42 (s, 1H),7.24–7.49 (m, 5H), 7.22 (m, J = 8.1 Hz, 2H), 7.20 (s, 1H), 7.16 (m, J = 8.1 Hz, 2H), 7.08 (d, J = 16.61 Hz, 1H), 6.61 (d, J = 16.61 Hz, 1 H), 5.08 (dd, J = 4.5, 2.0 Hz, 1H), 5.05 (br. d, J = 4.5 Hz, 1H), 2.20 (s, 3H).

6-[(*E*)-2-(4-methoxyphenyl)ethenyl]-4-phenyl-3,4-dihydropyrimidin-2(1*H*)-one (**3d**) [42]. Yellow powder; 75% yield; m.p. 207–209 °C. ^1^H NMR (400 MHz, DMSO-d_6_) δ 8.35 (s, 1H), 7.24–7.40 (m, 7H),7.21 (s, 1H), 7.03 (d, J = 16.61 Hz, 1H), 6.91 (m, J = 8.7 Hz, 2H), 6.47 (d, J = 16.61 Hz, 1H), 5.09 (dd, J = 4.5 Hz, 2.0, 1H), 4.99 (br. d, J = 4.5 Hz, 1H), 3.75(s, 3 H).

4-(4-chlorophenyl)-6-[(*E*)-2-(4-chlorophenyl)ethenyl]-3,4-dihydropyrimidin-2(1*H*)-one (**3e**). Pale yellow powder; 82% yield; m.p. 202–203 °C. ^1^H NMR (400 MHz, DMSO-d_6_) δ 8.37 (1H, s), 7.45 (2H, d, *J* = 8.7 Hz), 7.37–7.41 (2H, m), 7.36 (2H, d, *J* = 7.3 Hz), 7.28–7.33 (3H, m), 7.27 (1H, d, *J* = 2.0 Hz), 7.24 (1H, d, *J* = 3.7 Hz), 7.07 (1H, d, *J* = 16.6 Hz), 6.65 (1H, d, *J* = 16.6 Hz), 5.11 (1H, dd, *J* = 4.6, 2.1 Hz), 5.08 (1H, d, *J* = 4.7 Hz). ^13^C NMR (101 MHz, DMSO-*d*_6_) δ 152.99, 143.82, 135.45, 133.86, 132.09, 131.73, 128.68 (2C), 128.49(2C), 128.05 (2C), 127.99 (2C), 126.39, 123.01, 103.92, 54.37. MS (EI), m/z (%):346 [M]^+^ (68), 344 [M]^+^ (100), 235 (35), 233 (88), 207 (15),138 (18), 128 (17),101 (22), 75 (27).

### 3.3. Synthesis and Characterization of Spiro[chromane-2,4′-pyrimidin]-2′(3′H)-ones

A mixture of 6-styryl-4-aryldihydropyrimidin-2-ones **3** (1 mmol) with 1,3-benzenediols **4** (2 mmol) in CHCl_3_ (8 mL) with the addition of CH_3_COOH (1.6 mL; 27.2 mmol) and CH_3_SO_3_H (0.1554 mL; 2.4 mmol) was heated at 40 °C for 3 h (TLC control). Then it was cooled, and the precipitate formed was filtered off and washed with water. The precipitate was taken up in ethyl acetate (3–5 mL) and refluxed for 10 min. The hot solution was separated from the powder of isomer **6** remaining in the precipitate. Then this solution was cooled, and the precipitated crystals of isomer **5** were filtered off. Both powders of substances **6** and **5** were dried in air.

(2S*,4R*,6′R*)-7-hydroxy-4,6′-diphenyl-5′,6′-dihydro-1′*H*-spiro[chromane-2,4′-pyrimidin]-2′(3′*H*)-one (**5a**) [43]. White powder; 17% yield; m.p. 257–258 °C.^1^H NMR (400 MHz, DMSO-d_6_) δ 9.21 (br. s., 1H), 7.21–7.43 (m, 8H), 7.17 (d, J = 7.6 Hz, 2H), 6.93 (br. s., 1H), 6.40 (d, J = 8.1 Hz, 1H), 6.23–6.29 (m, 2H), 4.85 (dd, J = 12.2, 2.0 Hz, 1H), 4.61 (dd, J = 11.4, 5.9 Hz, 1H), 2.14–2.17 (m, 2H), 2.02–2.08 (m, 1H), 1.80 (t, J = 12.8 Hz, 1H).

(2S*,4R*,6′R*)-7-hydroxy-4,6′-diphenyl-5′,6′-dihydro-1′*H*-spiro[chromane-2,4′-pyrimidin]-2′(3′*H*)-one (**6a**) [43]. White powder; 25% yield; m.p. 265–266 °C. ^1^H NMR (400 MHz, DMSO-d_6_) δ 9.18 (br. s., 1H), 7.22–7.38 (m, 9 H), 7.17 (d, J = 7.6 Hz, 2H), 6.81 (br. s., 1H), 6.36 (d, J = 8.5 Hz, 1H), 6.31 (d, J = 2.4 Hz, 1H), 6.23(dd, J = 8.5, 2.4 Hz, 1H), 4.57 (dd, J = 12.2, 3.0 Hz, 1H), 4.10 (dd, J = 12.6, 5.7 Hz, 1H), 2.19–2.24 (m, 2H), 2.08 (dd, J = 13.2, 5.7 Hz, 1H), 1.81 (t, J = 12.6 Hz, 1H).

(2S*,4R*,6′R*)-4-(4-chlorophenyl)-7-hydroxy-6′-phenyl-5′,6′-dihydro-1′*H*-spiro[chromane-2,4′-pyrimidin]-2′(3′*H*)-one (**5b**) [43]. White powder; 18% yield; m.p. 279–281 °C. ^1^H NMR (500 MHz, DMSO-d_6_) δ 9.28 (s, 1H), 7.28–7.45 (m, 8H), 7.18 (d, J = 8.2 Hz, 2H), 7.01 (br. s., 1H), 6.38 (d, J = 8.8 Hz, 1H), 6.25–6.29 (m, 2H), 4.87 (dd, J = 12.5, 3.5 Hz, 1H), 4.65 (dd, J = 12.5, 5.8 Hz, 1H), 2.12–2.19 (m, 2H), 2.05 (t, J = 12.5 Hz, 1H), 1.81 (t, J = 12.5 Hz, 1H).

(2R*,4R*,6′R*)-4-(4-chlorophenyl)-7-hydroxy-6′-phenyl-5′,6′-dihydro-1′*H*-spiro[chromane-2,4′-pyrimidin]-2′(3′*H*)-one (**6b**) [43]. White powder; 52% yield; m.p. 278–279 °C. ^1^H NMR (500 MHz, DMSO-d_6_) δ 9.36 (br. s., 1 H), 7.54 (s, 1H), 7.39 (d, J = 7.5 Hz, 2H), 7.25–7.37 (m, 5H), 7.18 (d, J = 7.5 Hz, 2H), 7.01 (s, 1H), 6.31–6.36 (m, 2H), 6.24 (d, J = 7.8 Hz, 1H), 4.55 (dd, J = 10.5, 3.0 Hz, 1H), 4.15 (dd, J = 12.5, 5.6 Hz, 1H), 2.05–2.23 (m, 3H), 1.80 (t, J = 12.5 Hz, 1H).

(2R*,4R*,6′R*)-7-hydroxy-6′-phenyl-4-(p-tolyl)-5′,6′-dihydro-1′*H*-spiro[chromane-2,4′-pyrimidin]-2′(3′*H*)-one (**6c**) [43]. White powder; 36% yield; m.p. 227–229 °C. ^1^H NMR (400 MHz, DMSO-d_6_) δ 9.27 (br. s., 1H), 7.42 (br. s., 1H), 7.25–7.37 (m, 5H), 7.13 (d, J = 8.0 Hz, 2H), 7.04 (d, J = 8.0 Hz, 2H), 6.88 (br. s., 1H), 6.36 (d, J = 8.5 Hz, 1H), 6.27 (d, J = 1.5 Hz, 1H), 6.21 (dd, J = 8.5, 1.5 Hz, 1H), 4.54 (d, J = 12.5 Hz, 1H), 4.05 (dd, J = 13.0, 5.8 Hz, 1H), 2.29 (s, 3H), 2.12–2.21 (m, 2H), 2.07 (dd, J = 12.5, 5.8 Hz, 1H), 1.78 (t, J = 13.0 Hz, 1H).

(2R*,4R*,6′R*)-7-hydroxy-4-(4-methoxyphenyl)-6′-phenyl-5′,6′-dihydro-1′*H*-spiro[chromane-2,4′-pyrimidin]-2′(3′*H*)-one (**6d**) [43]. White powder; 18% yield; m.p. 212–214 °C. ^1^H NMR (400 MHz, DMSO-d_6_) δ 9.26 (br. s., 1H), 7.43 (br. s., 1H), 7.2–7.39 (m, 5H), 7.06 (d, J = 8.1 Hz, 2H), 6.86–6.92 (m, 3H), 6.37 (d, J = 8.6 Hz, 1H), 6.19–6.27 (m, 1H), 6.28 (s, 1H), 4.55 (dd, J = 12.1, 2.1 Hz, 1H), 4.04 (dd, J = 12.0, 5.4 Hz, 1H), 3.74 (s, 3H), 2.11–2.24 (m, 2H), 1.98–2.09 (m, 1H), 1.78 (t, J = 12.7 Hz, 1H).

(2S*,4R*,6′R*)-4-(4-Chlorophenyl)-7-hydroxy-8-methyl-6′-phenyl-5′,6′-dihydro-1′*H* spiro[chromane-2,4′-pyrimidin]-2′(3′*H*)-one (**5e**) [43]. White powder; 14% yield; m.p. 220–222 °C. ^1^H NMR (400 MHz, DMSO-d_6_) δ 9.10 (s, 1H), 7.28–7.43 (m, 7H), 7.25 (s, 1H), 7.18 (d, J = 8.1 Hz, 2H), 6.97 (s, 1H), 6.32 (d, J = 8.5 Hz, 1H), 6.23 (d, J = 8.5 Hz, 1H), 4.93 (dd, J = 12.7, 2.2 Hz, 1H), 4.63 (dd, J = 12.4, 6.1 Hz, 1H), 2.12–2.19 (m, 2H), 2.00–2.05 (m, 4H), 1.82 (t, J = 12.7 Hz, 1H).

(2S*,4R*,6′R*)-4,6′-bis(4-chlorophenyl)-7-hydroxy-5′,6′-dihydro-1′*H*-spiro[chromane-2,4′-pyrimidin]-2′(3′*H*)-one (**5f**). White powder; 25% yield; mp 208–209 °C. ^1^H NMR (400 MHz, DMSO-d_6_) δ9.30 (s, 1H), 7.45 (d, J = 8.7 Hz, 2H), 7.42 (d, J = 8.7 Hz, 2H), 7.38 (d, J = 8.5, 2H), 7.35 (s, 1H), 7.18 (d, J = 8.5 Hz, 2H), 7.07 (s, 1H), 6.38 (d, J = 8.6 Hz, 1H), 6.26 (dd, J = 8.6, 2.4Hz, 2H), 4.86 (dd, 1H, J = 12.6, 3.9 Hz, 1H), 4.64 (dd, J = 12.6, 6.1 Hz, 1H), 2.14 (m, 2H), 2.03 (t, J = 12.6 Hz, 1H), 1.78 (t, J = 12.6 Hz, 1H). ^13^C NMR (101 MHz, DMSO-d_6_) δ 157.01, 154.83, 152.74, 143.90, 141.36, 131.95, 131.03, 130.27 (2C), 129.57, 128.57 (4C), 128.43 (2C), 115.44, 108.61, 103.37, 81.99, 59.75, 41.90, 40.38, 34.97. IR (ν/cm^−1^): 3411 (OH), 3239 (NH), 1668 (C=O), 1488 (C=C), 1070 (C-O-C). MS (EI), m/z (%): 456 [M]^+^ (16), 454 [M]^+^ (10), 235 (19), 233 (100), 197 (28), 140 (28), 103 (14), 77 (13). Elemental analysis calcd (%) for C_24_H_20_Cl_2_N_2_O_3_: C, 63.31; H, 4.43; N, 6.15; found: C, 63.05; H, 4.41; N, 6.12.

(2S*,4R*,6′R*)-7,8-dihydroxy-4,6′-diphenyl-5′,6′-dihydro-1′*H*-spiro[chromane-2,4′-pyrimidin]-2′(3′*H*)-one (**5g**). White powder; 32% yield; mp 228–229 °C. ^1^H NMR (400 MHz, DMSO-d_6_) δ 8.62 (br. s, 1H), 8.01 (br. s, 1H), 7.35–7.45 (m, 4H), 7.25–7.35 (m, 3H), 7.22 (br.s, 2H), 7.16 (d, J = 7.8 Hz, 2H), 6.95 (s, 1H), 6.26 (d, J = 8.4 Hz, 1H), 5.88 (d, J = 8.4 Hz, 1H), 5.25 (dd, J = 12.3, 3.6, 1H), 4.62 (dd, J = 12.6, 6.1Hz, 1H), 1.95–2.17 (m, 3H), 1.79 (t, J = 12.5 Hz, 1H). ^13^C NMR (101 MHz, DMSO-d_6_) δ 155.02, 145.07, 144.08, 142.78, 141.17, 133.19, 133.01, 128.46, 127.35 (4C), 126.58 (3C), 126.38, 118.14, 116.81, 108.15, 82.17, 49.86, 42.61, 40.43, 35.76.IR (ν/cm^−1^): 3424 (OH), 3320, 3227 (NH), 1672 (C=O), 1613, 1493 (C=C), 1062 (C-O-C). MS (EI), m/z (%): 402 [M]^+^ (21), 214 (85), 189 (100), 146 (17), 128 (19), 104 (23), 77 (27). Elemental analysis calcd (%) for C_24_H_22_N_2_O_4_: C, 71.63; H, 5.51; N, 6.96; found: C, 71.48; H, 5.49; N, 6.93.

(2R*,4R*,6′R*)-7,8-dihydroxy-4,6′-diphenyl-5′,6′-dihydro-1′*H*-spiro[chromane-2,4′-pyrimidin]-2′(3′*H*)-one (**6g**). White powder; 29% yield; mp 251–252 °C. ^1^H NMR (400 MHz, DMSO-d_6_) δ 8.69 (br. s, 1H), 8.17 (br. s, 1H), 7.48 (br. s, 1H), 7.25–7.44 (m, 7H), 7.22 (t, J = 7.8 Hz, 1H), 7.16 (d, J = 7.8 Hz, 2H), 6.92 (br. s, 1H), 6.20 (d, J = 8.3 Hz, 1H), 5.83 (d, J = 8.3 Hz, 1H), 4.71 (dd, J = 12.6, 3.5 Hz, 1H), 4.10 (dd, J = 12.8, 5.9 Hz, 1H), 2.22 (t, J = 12.8 Hz, 1H), 2.13 (dd, J = 12.6, 3.5 Hz, 1H), 2.06 (dd, J = 12.8, 5.9 Hz, 1H), 1.75 (t, J = 12.6 Hz, 1H). ^13^C NMR (101 MHz, DMSO-d_6_) δ 155.45, 144.77, 144.41, 142.69, 142.08, 133.31, 128.47 (6C), 127.50, 126.60 (3C), 126.49, 118.32, 115.68, 108.21, 84.11, 50.71, 40.87, 37.91. MS (EI), m/z (%): 402 [M]^+^(4), 214 (47), 213 (80), 189 (100), 146 (12), 128 (11), 104 (15), 77 (18).

(2S*,4R*,6′R*)-4-(4-chlorophenyl)-7,8-dihydroxy-6′-phenyl-5′,6′-dihydro-1′*H*-spiro[chromane-2,4′-pyrimidin]-2′(3′*H*)-one (**5h**). White powder; 37% yield; mp 240–242 °C. ^1^H NMR (400 MHz, DMSO-d_6_) δ 8.64 (s, 1H), 8.01 (s, 1H), 7.32–7.43 (m, 7H), 7.29 (s, 1H), 7.18 (d, J = 8.1 Hz, 2H), 6.92 (s, 1H), 6.27 (d, J = 8.4 Hz, 1H), 5.87 (d, J = 8.4 Hz, 1H), 5.24 (d, J = 12.0 Hz, 1H), 4.64 (dd, J = 13.0, 6.6 Hz, 1H), 2.07–2.18 (m, 2H), 2.03 (t, J = 12.9 Hz, 1H), 1.79 (t, J = 13.0 Hz, 1H). ^13^C NMR (101 MHz, DMSO-d_6_) δ 155.04 144.25, 144.06, 142.72, 141.17, 133.26 (2C), 130.93, 128.46 (4C), 127.37, 126.58 (3C), 118.00, 116.36, 108.26, 82.15, 49.85, 42.55, 39.73, 35.24. IR (ν/cm^−1^): 3552 (OH), 3322, 3228 (NH), 1664 (C=O), 1629, 1599, 1504, 1481 (C=C), 1271, 1165, 1065 (C-O-C). MS (EI), m/z (%): 438 [M]^+^ (13), 436 [M]^+^ (36), 249 (47), 247 (35), 213 (67), 189 (100), 104 (13). Elemental analysis calcd (%) for C_24_H_21_ClN_2_O_4_: C, 65.98; H, 4.85; N, 6.41; found: C, 65.76; H, 4.82; N, 6.38.

(2R*,4R*,6′R*)-4-(4-chlorophenyl)-7,8-dihydroxy-6′-phenyl-5′,6′-dihydro-1′*H*-spiro[chromane-2,4′-pyrimidin]-2′(3′*H*)-one (**6h**). White powder; 38% yield; m.p. 263–264 °C. ^1^H NMR (400 MHz, DMSO-d_6_) δ 8.69 (br. s., 1H), 8.18 (br. s., 1H), 7.45 (br. s., 1H), 7.23–7.40 (m, 7H), 7.16 (d, J = 7.8 Hz, 2H), 6.92 (br. s., 1H), 6.23 (d, J = 8.30 Hz, 1H), 5.81 (d, J = 8.30 Hz, 1H), 4.71 (br.d, J = 12.5 Hz, 1H), 4.15 (dd, J = 12.8, 6.1 Hz, 1H), 2.03–2.22 (m, 3H), 1.75 (t, J = 12.8 Hz, 1H). ^13^C NMR (101 MHz, DMSO-d_6_) δ 155.39, 144.53, 143.83, 142.68, 142.06, 133.37, 131.00, 130.24 (2C), 128.47 (4C), 127.50, 126.59 (2C), 118.18, 115.24, 108.30, 84.09, 50.69, 40.69, 38.76, 37.88. MS (EI), m/z (%): 438 [M]^+^ (2), 436 [M]^+^ (6), 249 (53), 247 (38), 213 (64), 189 (100), 104 (15). 77 (23).

(2S*,4R*,6′R*)-7,8-dihydroxy-6′-phenyl-4-(p-tolyl)-5′,6′-dihydro-1′*H*-spiro[chromane-2,4′-pyrimidin]-2′(3′*H*)-one (**5i**). White powder; 16% yield; m.p. 235–236 °C. ^1^H NMR (400 MHz, DMSO-d_6_) δ 8.62 (s, 1H), 8.00 (s, 1H), 7.42 (d, J = 7.7 Hz, 2H), 7.38 (t, J = 7.7 Hz, 2H), 7.29 (t, J = 7.7 Hz 1H), 7.21 (s, 1H), 7.10 (d, J = 8.0 Hz, 2H), 7.04 (d, J = 8.0 Hz, 2H), 6.95 (br. s., 1H), 6.25 (d, J = 8.6 Hz, 1H), 5.88 (d, J = 8.6 Hz, 1H), 5.25 (dd, J = 12.0, 3.0 Hz, 1H), 4.56 (dd, J = 12.6, 6.0 Hz, 1H), 2.26 (s, 3H), 2.08–2.16 (m, 1H), 2.04–2.08 (m, 1H),1.98–2.03 (m, 1H), 1.78 (t, J = 12.70 Hz, 1H). ^13^C NMR (101 MHz, DMSO-d_6_) δ 155.09, 144.04, 142.78, 141.96, 141.16, 135.34, 133.16, 129.04 (2C), 128.46 (3C), 128.35, 127.36, 126.59 (2C), 118.16, 117.01, 108.12, 82.18, 49.86, 42.65, 40.75, 35.31, 20.66. IR (ν/cm^−1^): 3445 (OH), 3324, 3295 (NH), 1651 (C=O), 1608, 1496 (C=C), 1240, 1067 (C-O-C). MS (EI), m/z (%): 416 [M]^+^ (14), 228 (32), 213 (100), 189 (42), 152 (25), 126 (11), 104 (13), 77 (12). Elemental analysis calcd (%) for C_25_H_24_N_2_O_4_: C, 72.10; H, 5.81; N, 6.73; found: C, 71.86; H, 5.80; N, 6.70.

(2S*,4R*,6′R*)-7,8-dihydroxy-4-(4-methoxyphenyl)-6′-phenyl-5′,6′-dihydro-1′*H*-spiro[chromane-2,4′-pyrimidin]-2′(3′*H*)-one (**5j**). White powder; 17% yield; m.p. 191–192 °C. ^1^H NMR (400 MHz, DMSO-d_6_) δ 8.60 (s, 1H), 7.99 (s, 1H), 7.34–7.45 (m, 4H), 7.30 (br. s., 1H), 7.19 (br. s., 1 H), 7.07 (d, J = 8.5 Hz, 2H), 6.95 (br. s., 1H), 6.86 (d, J= 8.5 Hz, 2H), 6.25 (d, J = 8.3 Hz, 1H), 5.89 (d, J = 8.3 Hz, 1H), 5.24 (br.d, J = 12.0 Hz, 1H), 4.55 (dd, J = 12.5, 6.0 Hz, 1H), 3.72 (s, 3H), 1.98–2.21 (m, 3H), 1.78 (t, J = 12.8 Hz, 1H). ^13^C NMR (101 MHz, DMSO-d_6_) δ 157.80, 155.09, 144.03, 142.78, 141.13, 136.77, 133.14, 129.39 (2C), 128.45 (2C), 127.35, 126.58 (2C), 118.10, 117.22, 113.89 (2C), 108.09, 82.22, 55.00, 49.86, 42.67, 40.84, 34.86. IR (ν/cm^−1^): 3371 (OH), 3301 (NH), 1651 (C=O), 1509 (C=C), 1244, 1186 (C-O-C). MS (EI), m/z (%): 432 [M]^+^ (2), 306 (15), 229 (33), 244 (100), 213 (64), 189 (42), 126 (19), 77 (11). Elemental analysis calcd (%) for C_25_H_24_N_2_O_5_: C, 69.43; H, 5.59; N, 6.48; found: C, 69.36; H, 5.57; N, 6.45.

(2S*,4R*,6′R*)-4,6′-bis(4-chlorophenyl)-7,8-dihydroxy-5′,6′-dihydro-1′*H*-spiro[chromane-2,4′-pyrimidin]-2′(3′*H*)-one (**5k**). White powder; 34% yield; m.p. 242–243 °C. ^1^H NMR (400 MHz, DMSO-d_6_) δ 8.63 (s, 1H), 8.01 (s, 1H), 7.44 (s, 4H), 7.37 (d, J = 8.1 Hz, 2H), 7.25 (s, 1H), 7.18 (d, J = 8.1 Hz, 2H), 7.01 (s, 1H), 6.27 (d, J = 8.4 Hz, 1H), 5.86 (d, J = 8.4 Hz, 1H), 5.27 (dd, J = 12.3, 3.6 Hz, 1H), 4.65 (dd, J = 12.8, 6.1Hz, 1H), 2.07–2.16 (m, 2H), 2.03 (t, J = 12.8 Hz, 1H), 1.78 (t, J = 12.7 Hz, 1H). ^13^C NMR (101 MHz, DMSO-d_6_) δ 154.98, 144.22, 144.01, 141.66, 141.05, 133.22, 131.81, 130.94, 130.32 (2C), 128.55 (2C), 128.47 (2C), 128.42 (2C), 117.98, 116.32, 108.30, 82.12, 49.27, 42.24, 40.38, 35.11. IR (ν/cm^−1^): 3430 (OH), 3328, 3254 (NH), 1655 (C=O), 1599 (C=C), 1244, 1071 (C-O-C). MS (EI), m/z (%): 472 [M]^+^ (7), 470 [M]^+^ (11), 410 (5), 412 (8), 225 (23), 223 (100), 221 (13), 213 (98), 180 (24), 178 (13), 168 (15),166 (27), 140 (50), 138 (68), 127 (28), 111 (34), 102 (25), 77 (35). Elemental analysis calcd (%) for C_24_H_20_Cl_2_N_2_O_4_: C, 61.16; H, 4.28; N, 5.94; found: C, 60.87; H, 4.27; N, 5.93.

(2R*,4R*,6′R*)-4,6′-bis(4-chlorophenyl)-7,8-dihydroxy-5′,6′-dihydro-1′*H*-spiro[chromane-2,4′-pyrimidin]-2′(3′*H*)-one (**6k**). White powder; 28% yield; m.p. 249–250 °C. ^1^H NMR (400 MHz, DMSO-d_6_) δ 8.74 (s, 1H), 8.22 (s, 1H), 7.54 (br. s., 1H), 7.34–7.44 (m, 4H), 7.29 (d, J = 8.3 Hz, 2H), 7.15 (d, J = 8.3 Hz, 2H), 7.05 (s, 1H), 6.22 (d, J = 8.5 Hz, 1H), 5.81 (d, J = 8.5 Hz, 1H), 4.71 (d, J = 12.2 Hz, 1H) 4.14 (dd, J = 12.6, 6.1 Hz, 1H), 2.01–2.22 (m, 3H), 1.74 (t, J = 13.1 Hz, 1H). ^13^C NMR (101 MHz, DMSO-d_6_) δ 155.34, 144.58, 143.77, 142.65, 141.00, 133.34, 131.91, 131.03, 130.24 (2C), 128.56 (2C), 128.49 (2C), 128.41 (2C), 118.21, 115.21, 108.34, 84.03, 50.12, 40.62, 38.74, 37.60. MS (EI), m/z (%): 472 [M]^+^ (1), 470 [M]^+^ (3), 225 (18), 223 (100), 221 (15), 213 (82), 180 (21), 178 (13), 166 (23), 140 (45), 138 (70), 127 (28), 111 (36), 102 (27), 77 (45).

### 3.4. Single-Crystal X-Ray Structure Determination

Crystals for X-ray diffraction were obtained by slow evaporation of solutions of compound **5h** in DMF at room temperature in air.

Single-crystal X-ray diffraction experiments for compound **5h** were carried out using a SMART APEX2 CCD diffractometer (λ(Mo-Kα) = 0.71073 Å, graphite monochromator, ω-scans) at 100 K. Collected data were processed by the SAINT and SADABS programs incorporated into the APEX2 program package [46]. The structures were solved by the direct methods and refined by the full-matrix least-squares procedure against *F*^2^ in anisotropic approximation. Only one solvent DMF molecule can be localized and properly refined. Half of the DMF and half of the water molecules are strongly disordered and were eliminated from the refinement using the standard SQUEEZE option. The refinement was carried out with the SHELXTL program [47].

Crystallographic data for **5h**: C_24_H_21_N_2_O_4_Cl 1.5C_3_H_7_ON·0.5H_2_O are monoclinic, space group *C*2/*c*: *a* = 26.665(7) Å, *b* = 7.9790(13) Å, *c* = 26.546(7) Å, *β* = 107.004(8)°, *V* = 5401(2) Å^3^, *Z* = 8, *M* = 555.53, *d*_cryst_ = 1.366 g·cm^−3^. *wR*2 = 0.2296 calculated on *F*^2^*_hkl_* for all 5229 independent reflections with 2*θ* < 51.9°, (*GOF* = 1.046, *R* = 0.0981 calculated on *F_hkl_* for 2982 reflections with *I* > 2*σ*(*I*)).

Supplementary crystallographic data for this paper can be obtained free of charge from The Cambridge Crystallographic Data Centre via (http://www.ccdc.cam.ac.uk, accessed on 3 March 2025). CCDC numbers 2435296 (**5h**).

### 3.5. Biological Evaluation

#### 3.5.1. In Vitro Antibacterial Activity

All the synthesized compounds **5a**–**h** and **6a**–**h** were evaluated for their in vitro antimicrobial activity against a number of both Gram-positive and Gram-negative bacteria (*Staphylococcus aureus*, *Bacillus cereus*, *Enterococcus faecium*, *Micrococcus luteus*, *Escherichia coli*, *Pseudomonas fluorescence*) and yeast *Candida albicans*. Overnight cultures were grown at 37 °C in Lysogeny broth (LB) and diluted to obtain an opacity equivalent to 0.5 on the McFarland scale. Screening vials were filled with solutions of the test compounds in 0.5% DMSO as prepared above, with three replications for each treatment. Active pharmaceutical ingredient (API) pefloxacin (1–256 µg/mL) and 0.5% DMSO served as positive and negative controls, respectively. The entire vial was incubated at 35 ± 2 °C for 18 h. After incubation, the antibacterial activity of the test compounds was determined by measuring the absorption of the solution with a spectrophotometer at 500 nm [48].

#### 3.5.2. MIC Measurement

The MICs of the most active compounds were measured using the twofold serial broth dilution method. The test organisms were grown in suitable broth for 18 h at 37 °C. Twofold serial dilutions of solutions of the test compounds were prepared at 256, 128, 64, 32, 16, 8, 4, 2, 1 µg/mL. The tubes were then inoculated with the test microbe; each 5 mL received 0.1 mL of the above inoculum, and they were incubated at 37 °C. The vials were subsequently observed for the presence or absence of microbial growth. The MIC values of the prepared compounds are listed in Table 1.

#### 3.5.3. Hemolytic Activity Assay

The hemolytic effects of the most active compounds were measured using spectrophotometry (FlexA-200 Microplate Reader, Hangzhou Allsheng Instruments Co., Hangzhou, China) [49]. 1 milliliter of human blood was collected from human volunteers, added to a sterile, screw-top EDTA tube, and centrifuged at 850× *g* for 10 min. The upper layer was decanted, and the erythrocytes were rinsed several times with 10 mL cooled isotonic and sterile phosphate-buffered saline (PBS) with a pH of 7.4. The rinsed cells were resuspended in 2 mL sterile and cold PBS. Test compounds (256, 128, 64, 32, 16, 8, 4, 2, 1, 0,5, and 0.25 µg/mL) were added to the erythrocyte solution and incubated for 60 min at 37 °C. The absorbance of hemoglobin in the supernatant at 405 nm was used to calculate the hemolysis rate. A total of 0.1% Triton X-100 was used as a positive control, and PBS as the negative control. Hemolysis percentage was calculated using the following formula.

Hemolysis percentage = (Ab of sample − Ab of negative control)/Ab of positive control × 100

The data represented in Table 4 present the hemolytic activity of different compounds. Overall, all four compounds have less than 30% hemolysis activity, so all compounds are nontoxic to humans and safe.

#### 3.5.4. Biofilm Inhibition Assay

A broth culture prepared in brain-heart infusion broth (BHI) (37 °C, 16 h) was corrected to an OD_550_ of 0.8 (0.5 × 10^9^ to 1.0 × 10^9^ CFU/mL) and diluted 1:100. To evaluate the effect of the most active compounds in preventing biofilm formation, 200 μL of this suspension was added in each well of a TC-treated microplate (*Nunc*) with (1×, 2× and 4× MIC) or without the compound (control). Active pharmaceutical ingredient (API) pefloxacin was used as a positive control. After incubation at 37 °C for 24 h, planktonic cells were gently removed by washing twice with 200 μL PBS. After fixing samples (60 °C, 1 h), the biofilm biomass was quantified with crystal violet staining. Briefly, 200 μL of 10% (*w*/*v*) Hucker’s crystal violet was added to each well, and after incubation at room temperature for 5 min, each well was washed using tap water. After drying at 37 °C, 200 μL of 33% (*v*/*v*) glacial acetic acid was added to dissolve the stained dye for 15 min. The biofilm biomass was determined by measuring the absorbance at 492 nm using a FlexA-200 Microplate Reader. To evaluate the efficacy of compounds against preformed biofilms, 24-h biofilms were treated with 200 μL of broth with (2× and 4× MIC) or without (control) the selected compound at 37 °C for another 24 h and then washed with PBS [50,51].

### 3.6. Antioxidant Activity

Antioxidant activities of compounds 7 and 8 were measured by the 2,2-diphenyl-1-picryl-hydrazyl-hydrate (DPPH) radicals scavenging method, which was established by Blois (1958) and Brand-Williams et al. (1995) and adapted for 96-well plates [45]. The samples were dissolved in a DMSO: water mixture (50:450, *v*/*v* (µL/µL)), and dilutions were made with methanol (HPLC grade). Ascorbic acid was used as a positive control. The negative control was without any samples. 10 μL of 0.1 mM DPPH radical solution, freshly made in methanol, was added to 120 μL of samples or standards in 96-well plates. The plates were shaken for 1 min with the microplate reader (FlexA-200 Microplate Reader, China). They were incubated for 45 min in the dark at room temperature at 517 nm. % of DPPH scavenging activity was calculated according to % DPPH Scav. Act. = [(AControl ASample)/AControl] ×100. Statistical evaluation of data was performed using a student’s paired t-test. Data were expressed as mean ±standard deviation (mean ± SD) of experiments. The experiment was repeated three times (*n* = 3). The data were analyzed by Microsoft Office Excel and analyzed by an analysis of variance (*p* < 0.05).

### 3.7. Pharmacokinetic Parameters

Additionally, ADMET properties of newly designed compounds **5f**, **5b**, **5h**, and **6h** were predicted through SwissADME (http://www.swissadme.ch, accessed on 5 May 2025), which was utilized to evaluate the synthetic accessibility of the newly developed compounds. Computer-aided ADME studies were performed using the webserver pkCSM (http://biosig.unimelb.edu.au/pkcsm/prediction, accessed on 5 May 2025) to estimate the pharmacokinetic features of the prepared in-house compounds. StopTox (https://stoptox.mml.unc.edu/, accessed on 3 March 2025)online servers were used to access the toxicity analysis of new spyrochromanes [52].

## 4. Conclusions

A method for obtaining diastereomerically pure spiro[chromane-2,4′-pyrimidin]-2′(3′*H*)-ones by acid-catalyzed reaction of resorcinols with styryldihydropyrimidines was improved. Possibilities for controlling the stereoselectivity of the reaction were demonstrated: carrying out the reaction in a medium of chloroform and methanesulfonic acid facilitates the isolation of diastereomer **5**, while carrying out the reaction in the presence of toluenesulfonic acid makes it possible to isolate 100% isomer **6**. The antibacterial properties of diastereomeric pairs were studied individually on a panel of 8 bacterial culture strains and 1 fungal strain. The importance of isolating individual diastereoisomers was confirmed by the different antibacterial activity of diastereoisomers. The isomers with the axial conformation of the chromane and dioxopyrimidine rings showed the highest activity. Four promising compounds, **5f**, **5h**, **6h**, and **5b,** with high antibacterial activity, potential for further pharmaceutical development, have been identified.

A possible mechanism of action of these molecules as cell wall inhibitors has been suggested. Hemolytic and antiaggregatory activity of hit compounds has been studied. No toxic effect in the therapeutic concentration range has been shown.

Two hit—compounds (2S*,4R*,6′R*)-4-(4-chlorophenyl)-7-hydroxy-6′-phenyl-5′,6′-dihydro-1′*H*-spiro[chromane-2,4′-pyrimidin]-2′(3′*H*)-one (**5b**), (2S*,4R*,6′R*)-4,6′-bis(4-chlorophenyl)-7-hydroxy-5′,6′-dihydro-1′*H*-spiro[chromane-2,4′-pyrimidin]-2′(3′*H*)-one (**5f**) showed high level of antioxidant activity compared with ascorbic acid.

The calculated pharmacokinetic parameters of the hit compounds indicate good potential of these molecules as drug candidates capable of binding to blood proteins and penetrating the blood-brain barrier.

These findings establish the spirocyclic chromanopyrimidine scaffold—readily synthesized from aldehydes, polyphenols, and Biginelli adducts—as a promising chemotype for further medicinal chemistry optimization toward safer and more potent agents against infectious diseases and oxidative stress-related disorders.

## Figures and Tables

**Table 1:** The Scope and Isolated yields of compounds 5 and 6, %.

Isolated Yield of Compounds 5 and 6, %		
R_1_, R_2_, R_3_	Isomer 5	Isomer 6
**a**: R_1_ = R_2_ = R_3_ = H	17	25
**b**: R_1_ = H, R_2_ = Cl, R_3_ = H	18	52
**c**: R_1_ = H, R_2_ = Me, R_3_ = H	-	36
**d**: R_1_ = H, R_2_ = OMe, R_3_ = H	-	18
**e**: R_1_ = H, R_2_ = Cl, R_3_ = 2-Me	14	-
**f**: R_1_ = R_2_ = Cl, R_3_ = H	25	-
**g**: R_1_ = R_2_ = H, R_3_ = 2-OH	32	29
**h**: R_1_ = H, R_2_ = Cl, R_3_ = 2-OH	37	38
**i**: R_1_ = H, R_2_ = Me, R_3_ = 2-OH	16	-
**j**: R_1_ = H, R_2_ = MeO, R_3_ = 2-OH	17	-
**k**: R_1_ = R_2_ = Cl, R_3_ = 2-OH	34	28

**Table 2:** Optimization of the reaction condensation of 6-stiryl-4-aryldihydropyrimidin-2-one **3b** with the pyrogallol **4c**.

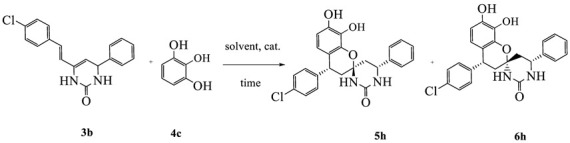					
Entry	Catalyst (mmol)	Promoter (mmol)	Time, h	Isolated Yield, %	Ratio 5h/6h, %
1	CH_3_SO_3_H (0.48)	AcOH (27.2)	3	47	50:50
2	CH_3_SO_3_H (1.2)	AcOH (27.2)	3	53	50:50
**3**	**CH_3_SO_3_H (2.4)**	**AcOH (27.2)**	**3**	**75**	**50:50**
4	CH_3_SO_3_H (3.6)	AcOH (27.2)	3	60	50:50
5	CH_3_SO_3_H (4.8)	AcOH (27.2)	3	43	50:50
6	CH_3_SO_3_H (7.2)	AcOH (27.2)	3	26	50:50
7	CH_3_SO_3_H (2.4)	-	3	42	94:6
8	CH_3_SO_3_H (2.4)	AcOH (6.8)	3	47	80:20
9	CH_3_SO_3_H (2.4)	AcOH (13.6)	3	55	63:37
10	CH_3_SO_3_H (2.4)	AcOH (40.8)	3	50	11:89
11	CH_3_SO_3_H (2.4)	AcOH (54.4)	3	44	7:93
12	CH_3_SO_3_H (2.4)	AcOH (54.4)	1	11	100:0
13	TsOH∙H_2_O (0.3)	AcOH (27.2)	3	-	-
14	CH_3_SO_3_H (1.2), TsOH∙H_2_O (1.2)	-	3	32	0:100
15	TsOH∙H_2_O (0.3)	-	3	-	-

Conditions: compound **3b** (1 mmol), compound **4c** (2 mmol), solvent CHCl_3_ (8 mL), T = 40 °C.

**Table 3:** The MIC values for a range of spiro[chromane-2,4′-pyrimidin]-2′(3′*H*)-ones **5** and **6** against several bacterial strains and fungi, µg/mL *.

ID	Bacterial Strain								Fungi
	Gram “+”					Gram “−”			
	*Staphylococcus* *aureus*, clinical Isolate	*Staphylococcus* *aureus ATCC-25923,* мкг/мл	*Bacillus cereus* IP 5832,	*Enterococcus faecium* K-1	*Micrococcus luteus 2665*	*Escherichia coli* C1,	*Escherichia coli* ADH52REF	*Pseudomonas fluorescens* A1	*Candida albicans* ATCC 10231,
**5a**	>256	32	16	256	16	>256	2	>256	64
**6a**	>256	>256	>256	>256	>256	>256	>256	>256	>256
**5b**	128	8	16	16	16	>256	8	8	32
**6b**	>256	>256	>256	>256	>256	>256	>256	>256	>256
**6c**	>256	>256	>256	>256	>256	>256	2	>256	>256
**6d**	>256	>256	>256	>256	>256	>256	>256	>256	>256
**5e**	256	64	64	256	64	>256	2	>256	32
**5f**	≥256	4	4	≥256	2	≥256	4	4	4
**5g**	>256	32	16	256	16	>256	2	>256	64
**6g**	128	32	128	128	128	>256	128	128	128
**5h**	16	16	16	8	8	>256	16	16	32
**6h**	64	16	8	16	8	>256	8	8	16
**5i**	≥256	8	8	≥256	16	≥256	4	4	4
**5j**	128	128	128	128	128	>256	16	>256	128
**5k**	≥256	≥256	≥256	≥256	4	>256	4	4	4
**6k**	≥256	≥256	≥256	≥256	16	>256	16	16	4
*Pef*. **	2	2	1	2	2	2	2	2	2

* Measurements performed in triplicate. ** *Pef.*: API Pefloxacin, as positive control.

**Table 4:** Results of Hemolytic Activity of compounds **5b**, **5f**, **5h**, **6h** at a concentration of 32 µg/mL.

No.	Compound	Hemolytic Activity (%)	*p*-Value
1	**5b**	8.01 ± 0.16	<0.05
2	**5f**	12.31 ± 0.16	<0.05
3	**5h**	18.3 ± 0.10	<0.05
4	**6h**	31.51 ± 0.11	<0.05
5	Triton X-100 (Control)	95.59 ± 0.26	<0.05

**Table 5:** Predicted pharmacokinetic properties for the compounds (**5b**, **5f**, **5h**, **6h**) by SwissADME server.

Compd.	GI Absorption	Skin Permeation (cm/s)	BBB Permeation	P-gp Substrate	CYP1A2 Inhibitor	CYP2C19 Inhibitor	CYP2C9 Inhibitor	CYP2D6 Inhibitor	CYP3A4 Inhibitor
**5b**	High	−6.21	✓	✓	×	✓	✓	✓	✓
**5f**	High	−6.55	✓	✓	×	✓	✓	✓	×
**5h**	High		×	✓	×	✓	✓	×	×
**6h**	High	−6.38	×	✓	×	✓	✓	×	✓

**Table 6:** Antioxidant effect of compounds **5b**, **5f**, **5h**, **6h.**

No.	Compound	IC50 (μg /mL)
1	**5b**	>400
2	**5f**	>400
3	**5h**	12.5 ± 0.2
4	**6h**	12.5 ± 0.2
5	Ascorbic acid	12.5 ± 0.2

IC50 values expressed are means ± S.D. of three measurements.

## Data Availability

Data is contained within the article and supporting materials. In addition, CIFs are openly available at www.ccdc.cam.ac.uk/data_request/cif (accessed on 2 April 2025).

## Supplementary Materials

The following supporting information can be downloaded at: https://www.mdpi.com/article/10.3390/molecules30142954/s1, and contain copies of NMR spectra for products **3**, **5**, **6** and computational ADME data of spiro[chromane-2,4′-pyrimidin]-2′(3′*H*)-ones from SwissADME© server.

## Abbreviations

MIC Minimal Inhibition; API Active Pharmaceutical Ingredient.
