# Supplementary material for: Diastereoselective Synthesis and Biological Evaluation of Spiro[chromane-2,4′-pyrimidin]-2′(3′H)-ones as Novel Antimicrobial and Antioxidant Agents
Source: Molecules. 2025 Jul 14;30(14):2954. doi: 10.3390/molecules30142954 (PMC12300028; doi:10.3390/molecules30142954)
Supplement: Supplementary file 1 [file molecules-30-02954-s001.zip › molecules-3722510-supplementary.pdf]

Supplementary Information

Diastereoselective Synthesis and Biological Evaluation of Spi-ro[chromane-2,4′-pyrimidin]-2′(3′H)-ones as Novel Antimicrobial and Antioxidant Agents

Alena S. Karandeeva 1, Natalia A. Bogdanova 1, Mariya V. Kabanova 1, Sergey I. Filimonov 1, Zhanna V. Chirkova 1, Anna A. Romanycheva 2, Valeria A. Panova 2, Anton A. Shetnev 2,3,*, Nurila A. Togyzba-yeva 4,*, Saken A. Kanzhar 4, Nurbol O. Appazov 4 and Kyrill Yu. Suponitsky 5

1 Institute of Chemistry and Chemical Technology, Yaroslavl State Technical University, Moskovskii Prosp. 88, 150023 Yaroslavl, Russia
mariya_vk02@mail.ru (M.V.K.); filimonovsi@ystu.ru (S.I.F.), chirkovazhv@ystu.ru (Z.V.C.)
2 Pharmaceutical Technology Transfer Centre, Yaroslavl State Pedagogical University Named After K.D. Ushinsky, Respublikanskaya Str. 108, 150000 Yaroslavl, Russia
3 Moscow Center for Advanced Studies, Kulakova Str. 20, 123592 Moscow, Russia
4 Korkyt Ata Kyzylorda University, Aiteke Bi Str., 29A, 120014 Kyzylorda, Kazakhstan
5 A. N. Nesmeyanov Institute of Organoelement Compounds, Russian Academy of Sciences, Build. 1, Vavilova Str. 28, 119991 Moscow, Russia
* Correspondence: a.shetnev@list.ru (A.A.S.); nurila2009@mail.ru (N.T.)

Table of Content

S1. ^{1}H, ^{13}C NMR SPECTRA OF 6-STIRYL-4-ARYLDIHYDROPYRIMIDIN-2-ONES 3
S2. ^{1}H, ^{13}C NMR, NOESY SPECTRA OF SPIRO[CHROMANE-2,4'-PYRIMIDIN]-2'(3'*H*)-ONES 8
S3. COMPUTATIONAL ADME DATA OF SPIRO[CHROMANE-2,4'-PYRIMIDIN]-2'(3'*H*)-ONES FROM SWISSADME© SERVER 27

S1. ^{1}H, ^{13}C NMR spectra of 6-stiryl-4-aryldihydropyrimidin-2-ones

^{1}H NMR spectra of 4-phenyl-6-[(*Z*)-2-phenylethenyl]-3,4-dihydropyrimidin-2(1*H*)-one **(3a)**.

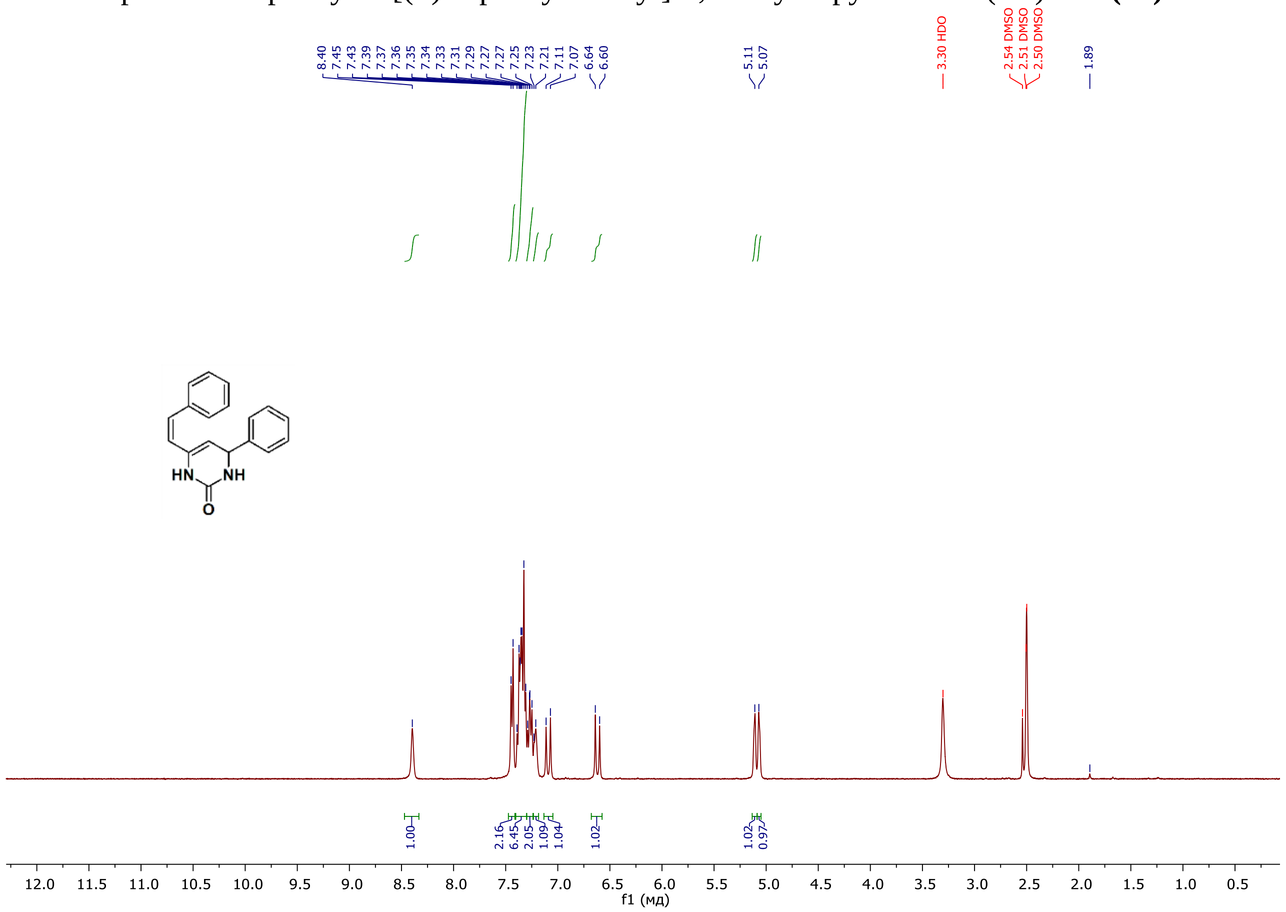

^{1}H NMR spectra of 6-[(*Z*)-2-(4-chlorophenyl)ethenyl]-4-phenyl-3,4-dihydropyrimidin-2(1*H*)-one **(3b)**.

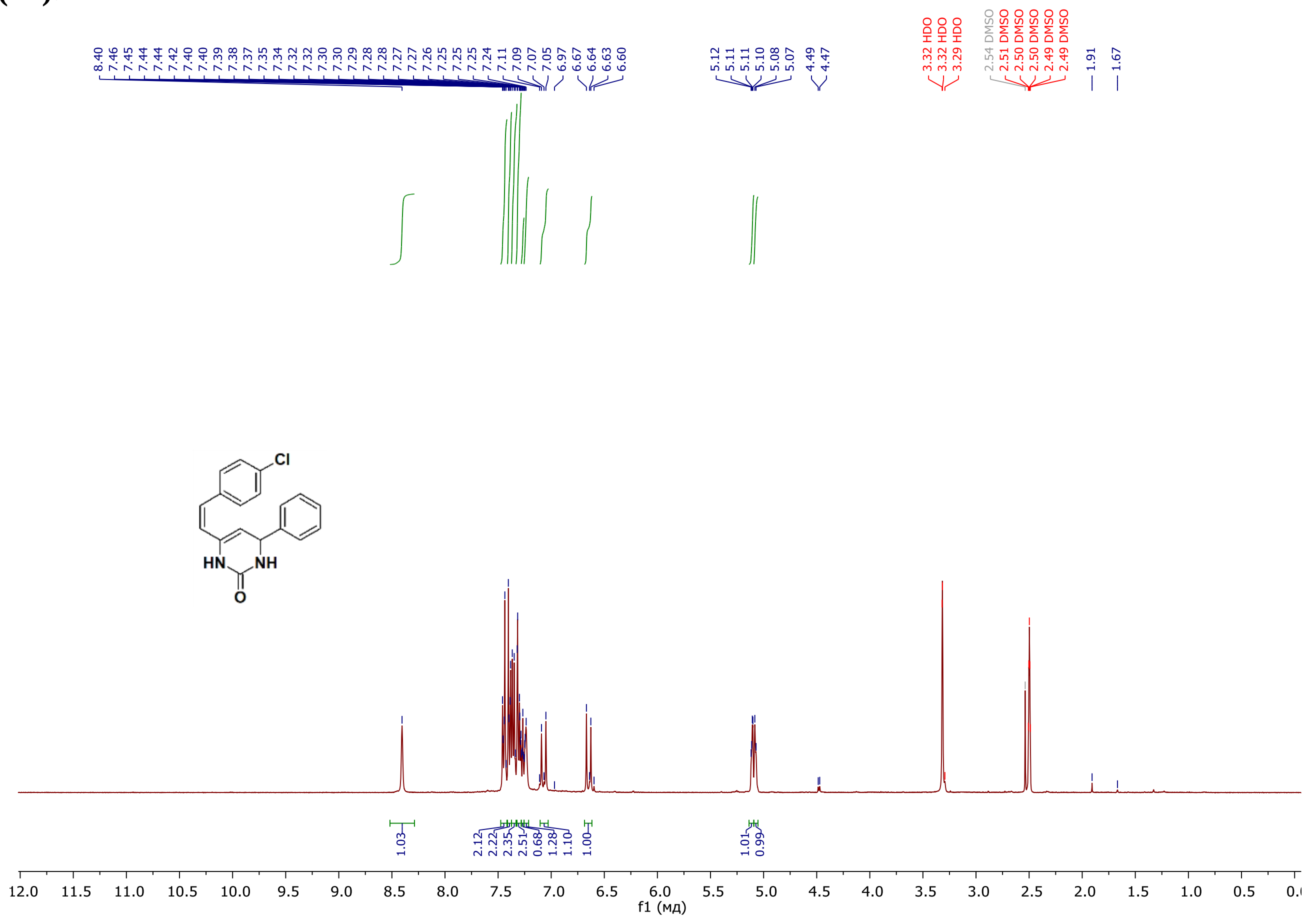

^{1}H NMR spectra of 6-[(*Z*)-2-(4-methylphenyl)ethenyl]-4-phenyl-3,4-dihydropyrimidin-2(1*H*)-one **(3c)**.

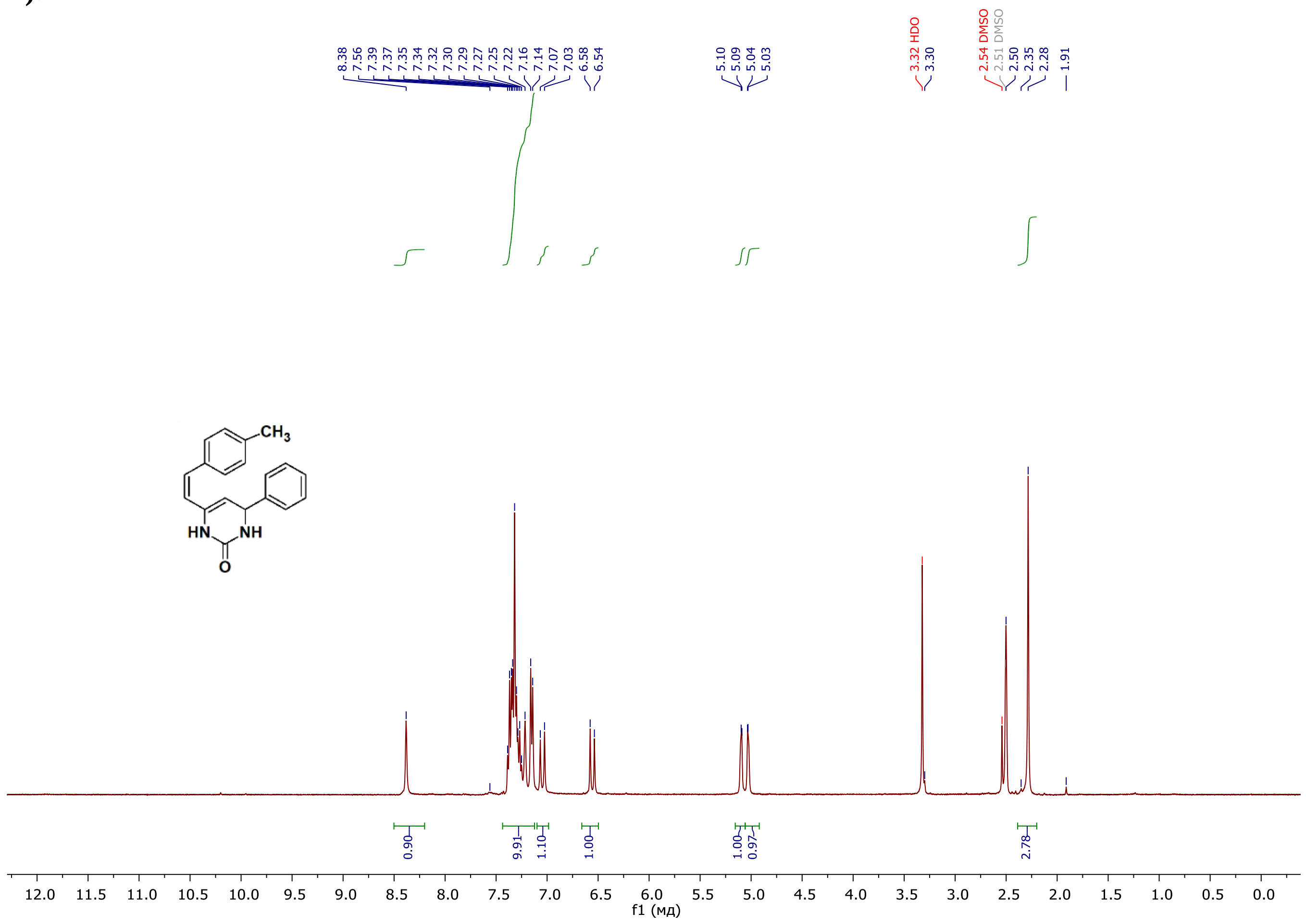

^{1}H NMR spectra of 6-[(*Z*)-2-(4-methoxyphenyl)ethenyl]-4-phenyl-3,4-dihydropyrimidin-2(1*H*)-one **(3d)**.

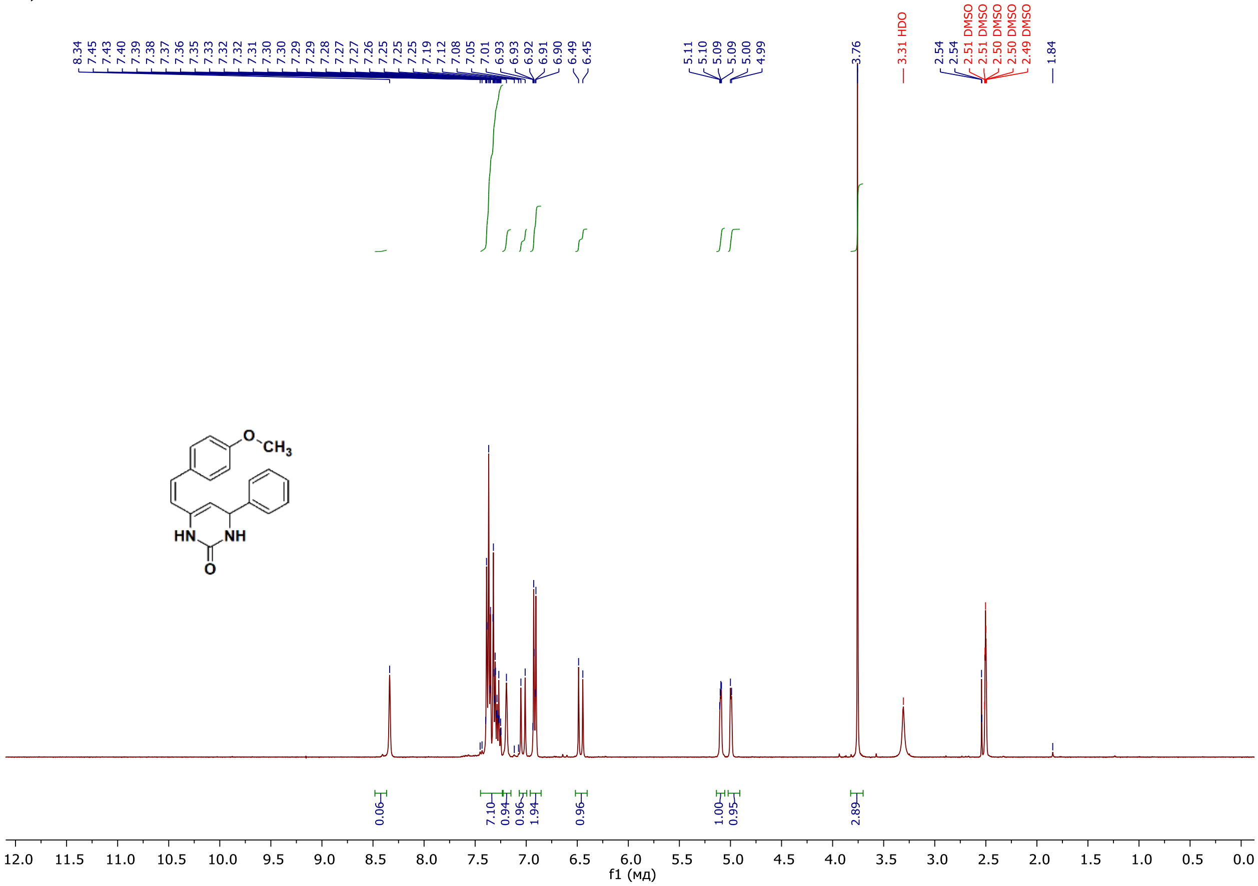

^{1}H and ^{13}C spectra of 4-(4-chlorophenyl)-6-[(*Z*)-2-(4-chlorophenyl)ethenyl]-3,4-dihydropyrimidin-2(1*H*)-one (**3e**)

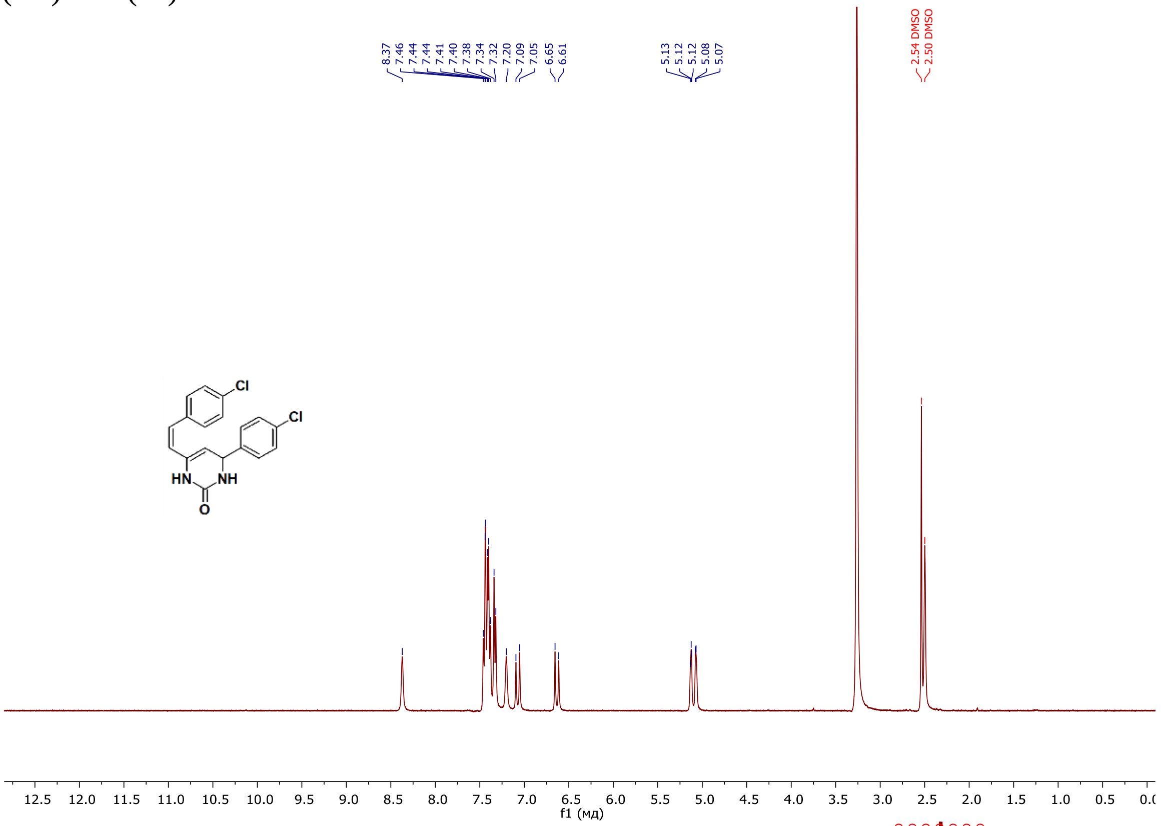

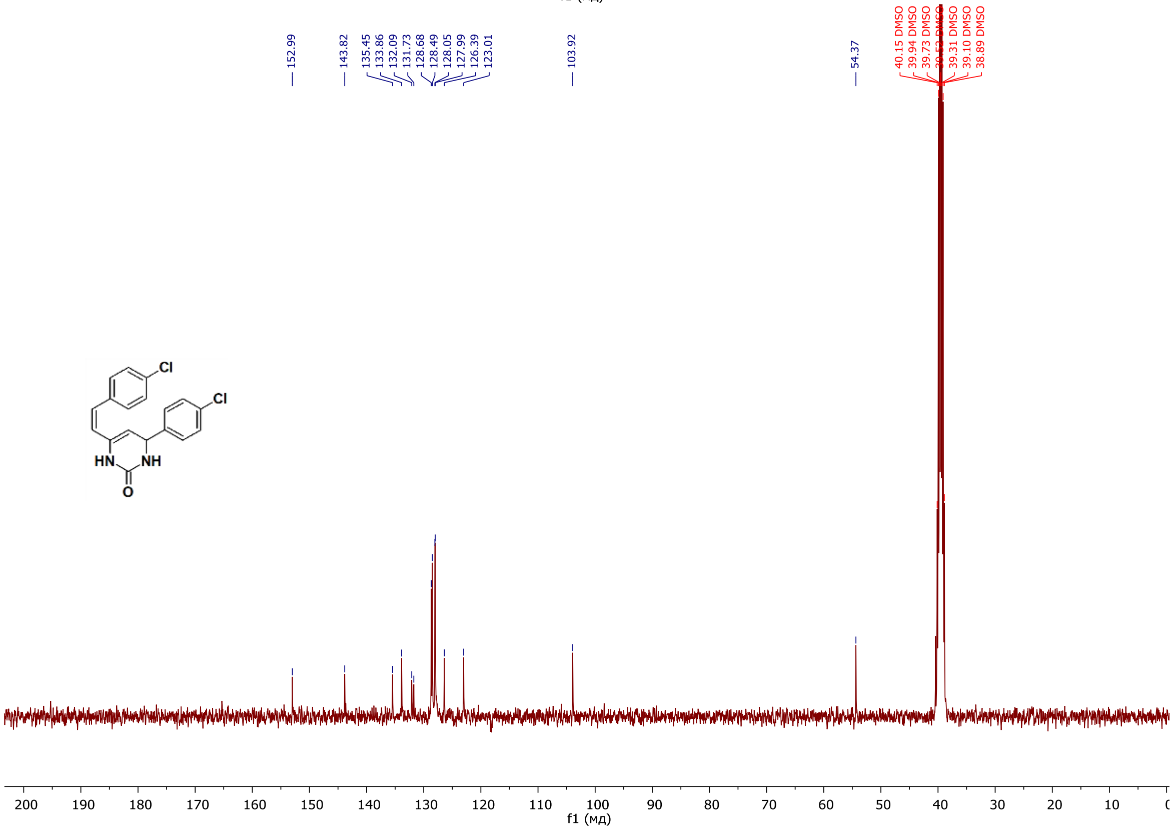

S2. ¹H, ¹³C NMR, NOESY spectra of spiro[chromane-2,4'-pyrimidin]-2'(3'*H*)-ones

¹H NMR spectra of (2*S**,4*R**,6'*R**)-7-hydroxy-4,6'-diphenyl-5',6'-dihydro-1'*H*-spiro[chromane-2,4'-pyrimidin]-2'(3'*H*)-one **(5a)**.

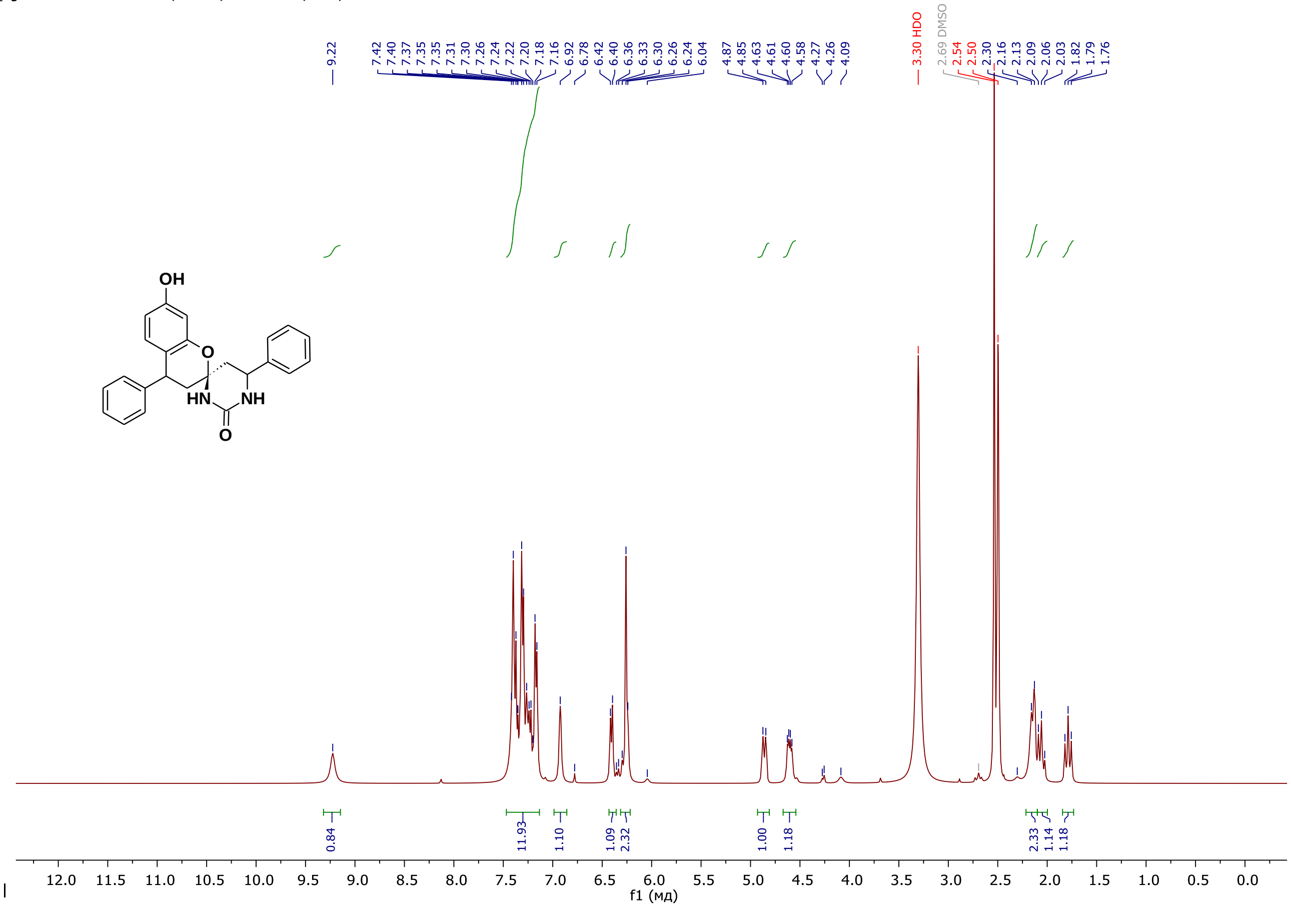

^{1}H NMR spectra of(2*R**,4*R**,6'*R**)-7-hydroxy-4,6'-diphenyl-5',6'-dihydro-1'*H*-spiro[chromane-2,4'-pyrimidin]-2'(3'*H*)-one**(6a)**

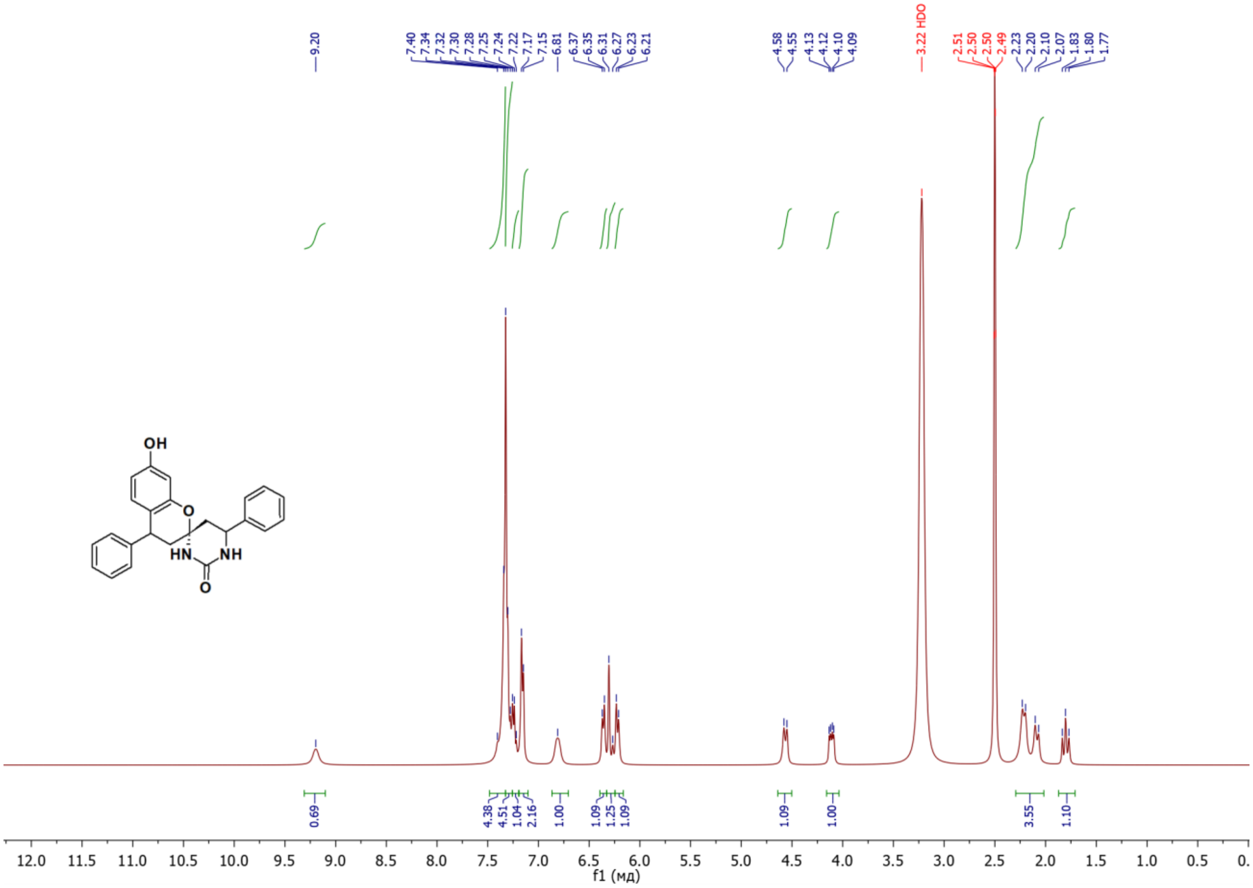

^{1}H NMR spectra of (2*S**,4*R**,6'*R**)-4-(4-chlorophenyl)-7-hydroxy-6'-phenyl-5',6'-dihydro-1'*H*-spiro[chromane-2,4'-pyrimidin]-2'(3'*H*)-one (**5b)**

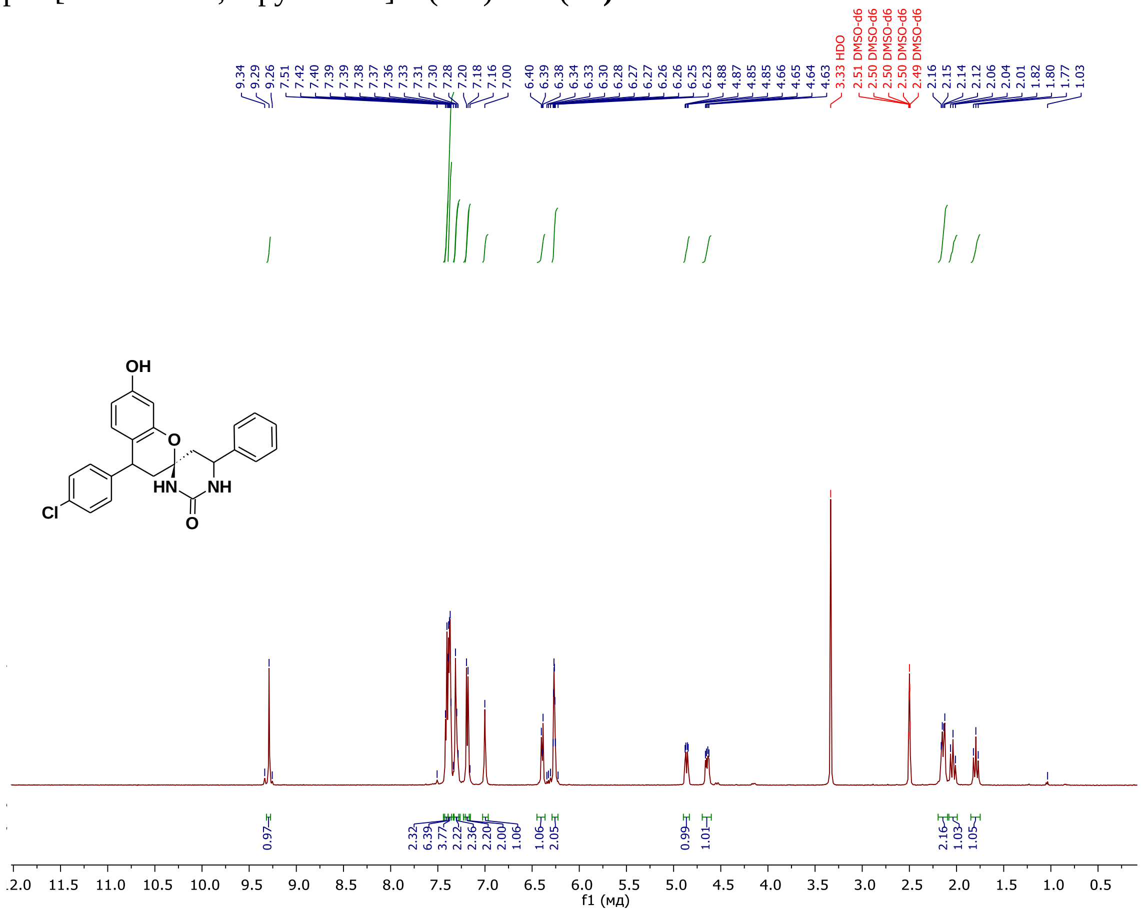

^{1}H NMR spectra of(2*R**,4*R**,6'*R**)-4-(4-chlorophenyl)-7-hydroxy-6'-phenyl-5',6'-dihydro-1'*H*-spiro[chromane-2,4'-pyrimidin]-2'(3'*H*)-one**(6b)**

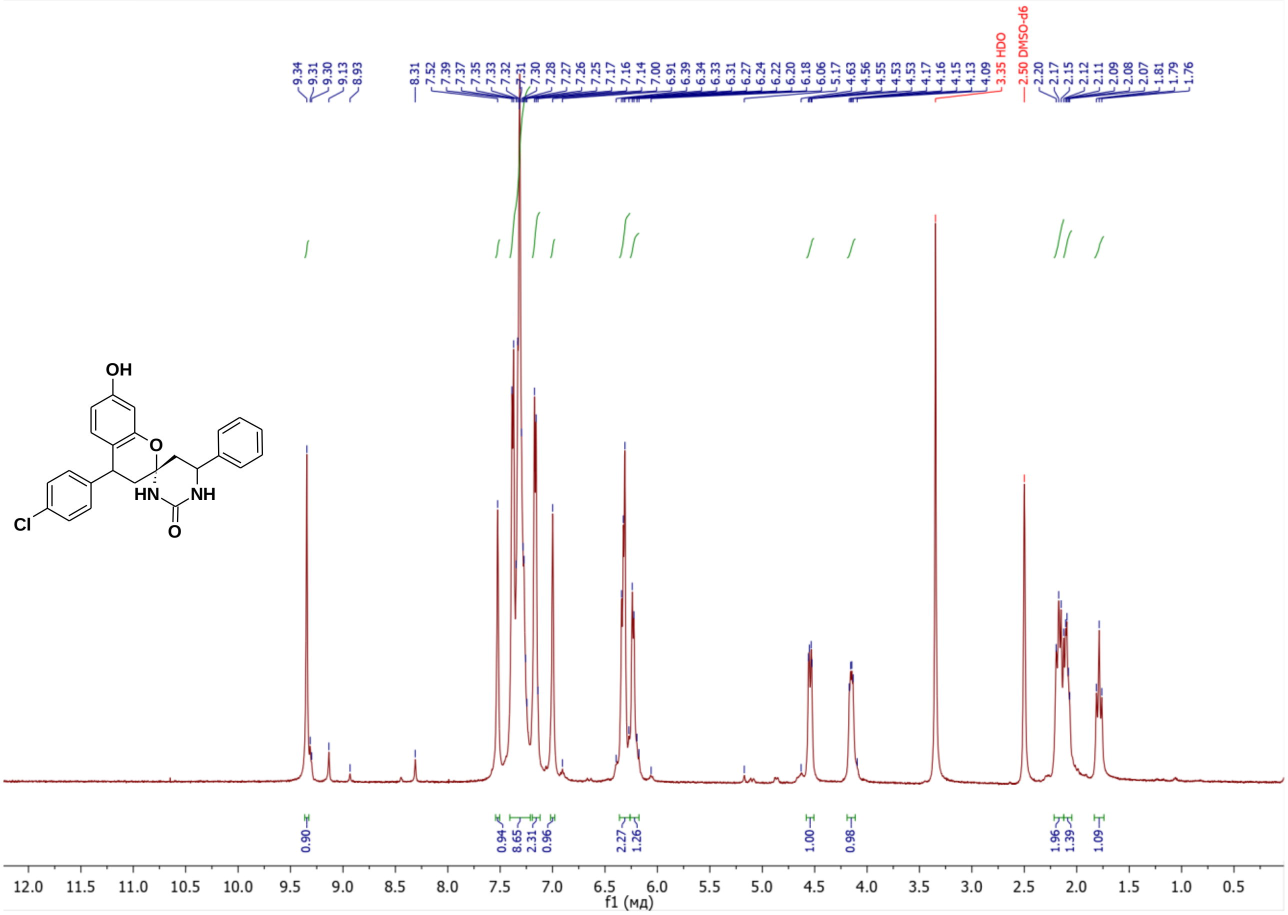

^{1}H NMR spectra of(2*R**,4*R**,6'*R**)-7-hydroxy-6'-phenyl-4-(*p*-tolyl)-5',6'-dihydro-1'*H*-spiro[chromane-2,4'-pyrimidin]-2'(3'*H*)-one **(6c)**

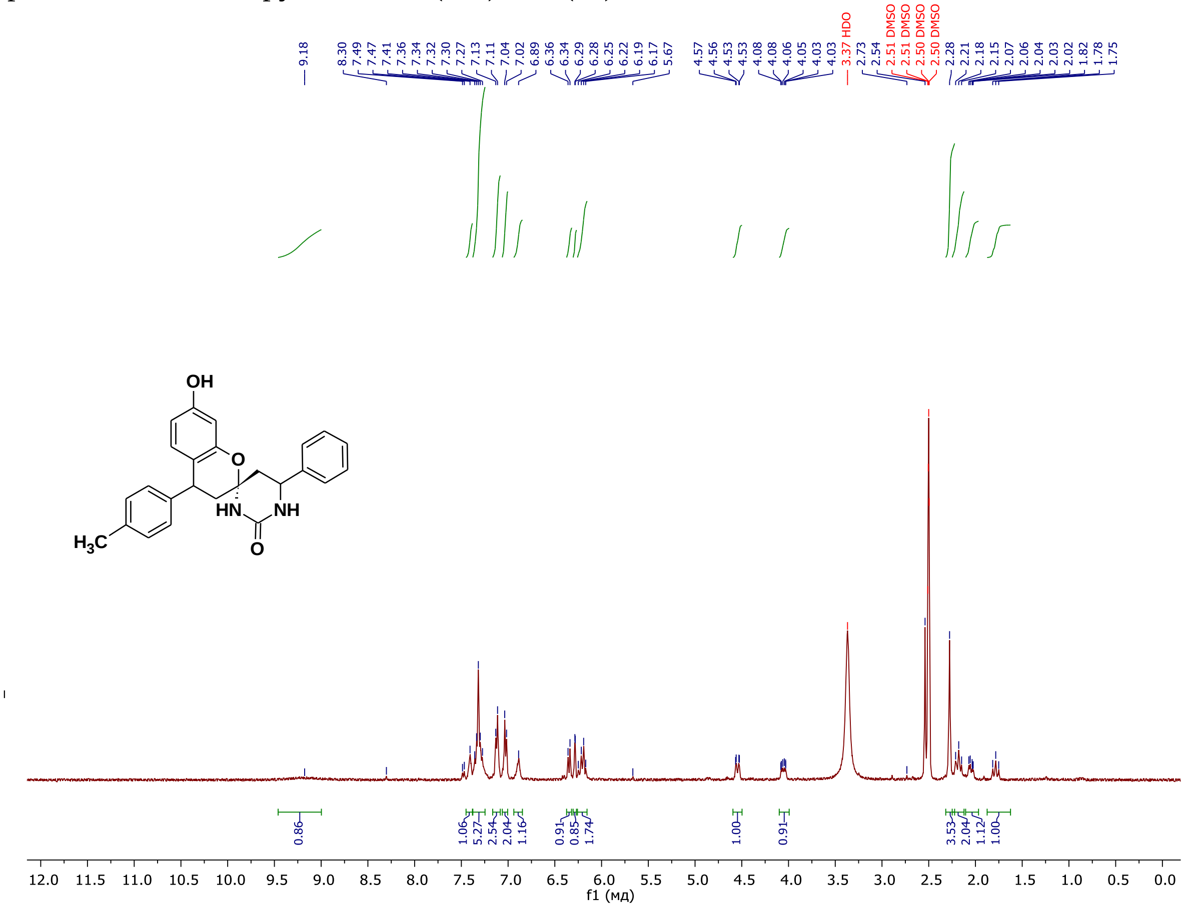

^{1}H NMR spectra of(2*R**,4*R**,6'*R**)-7-hydroxy-4-(4-methoxyphenyl)-6'-phenyl-5',6'-dihydro-1'*H*-spiro[chromane-2,4'-pyrimidin]-2'(3'*H*)-one **(6d)**

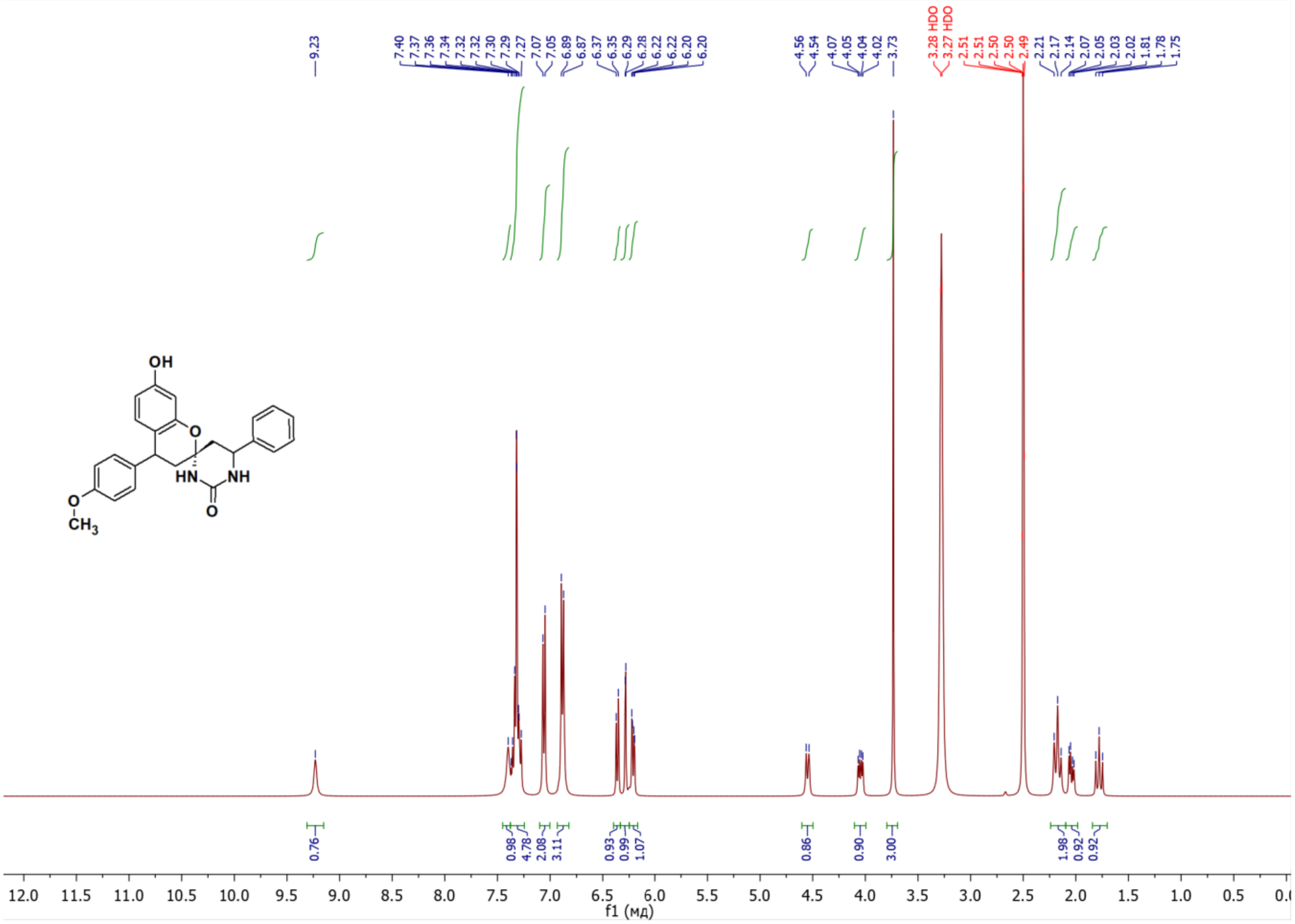

^{1}H NMR spectra of(2*S**,4*R**,6'*R**)-4-(4-chlorophenyl)-7-hydroxy-8-methyl-6'-phenyl-5',6'-dihydro-1'*H*-spiro[chromane-2,4'-pyrimidin]-2'(3'*H*)-one **(5e)**

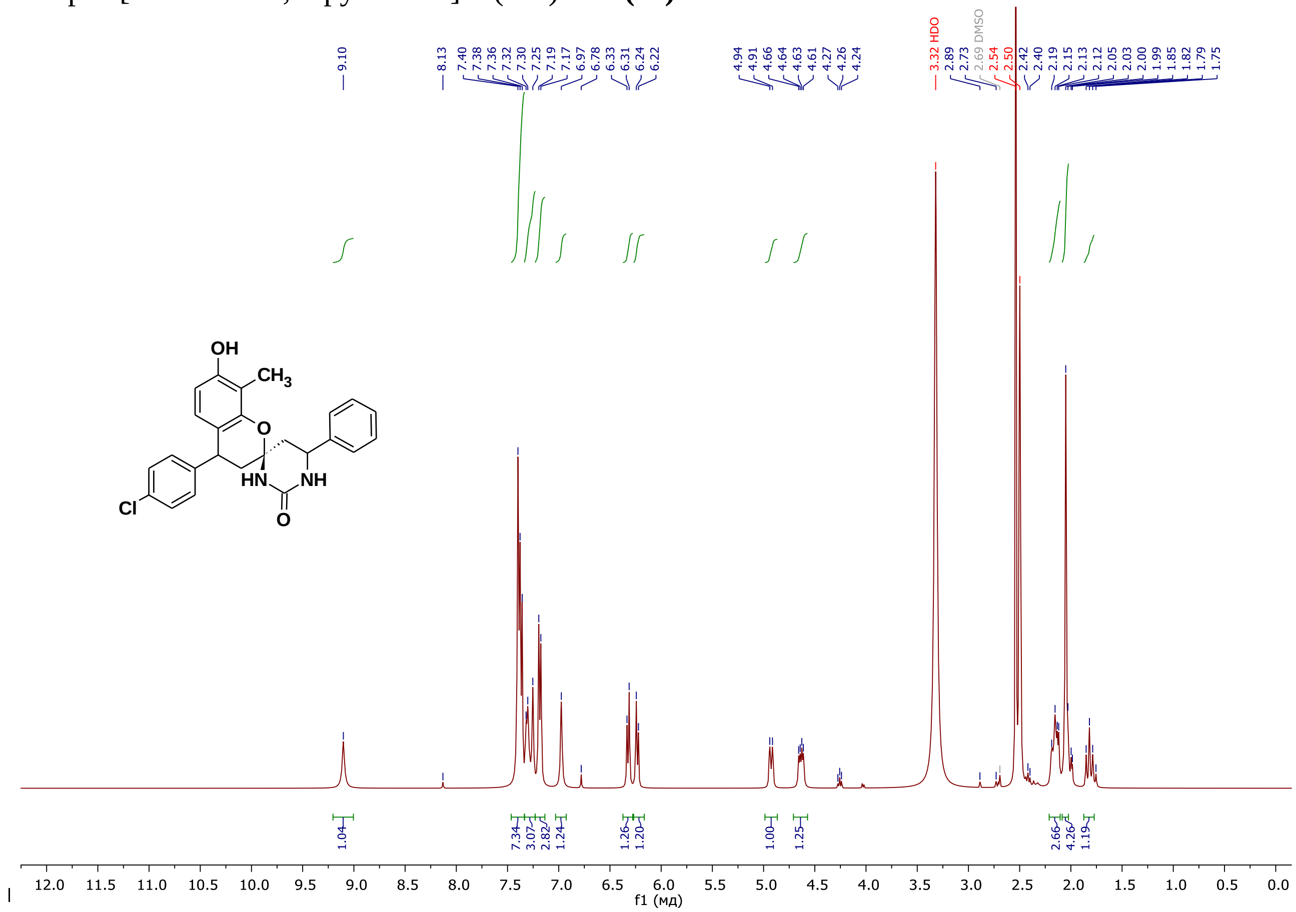

^{1}H and ^{13}C spectra of (2*S**,4*R**,6'*R**)-4,6'-bis(4-chlorophenyl)-7-hydroxy-5',6'-dihydro-1'*H*-spiro[chromane-2,4'-pyrimidin]-2'(3'*H*)-one (**5f**)

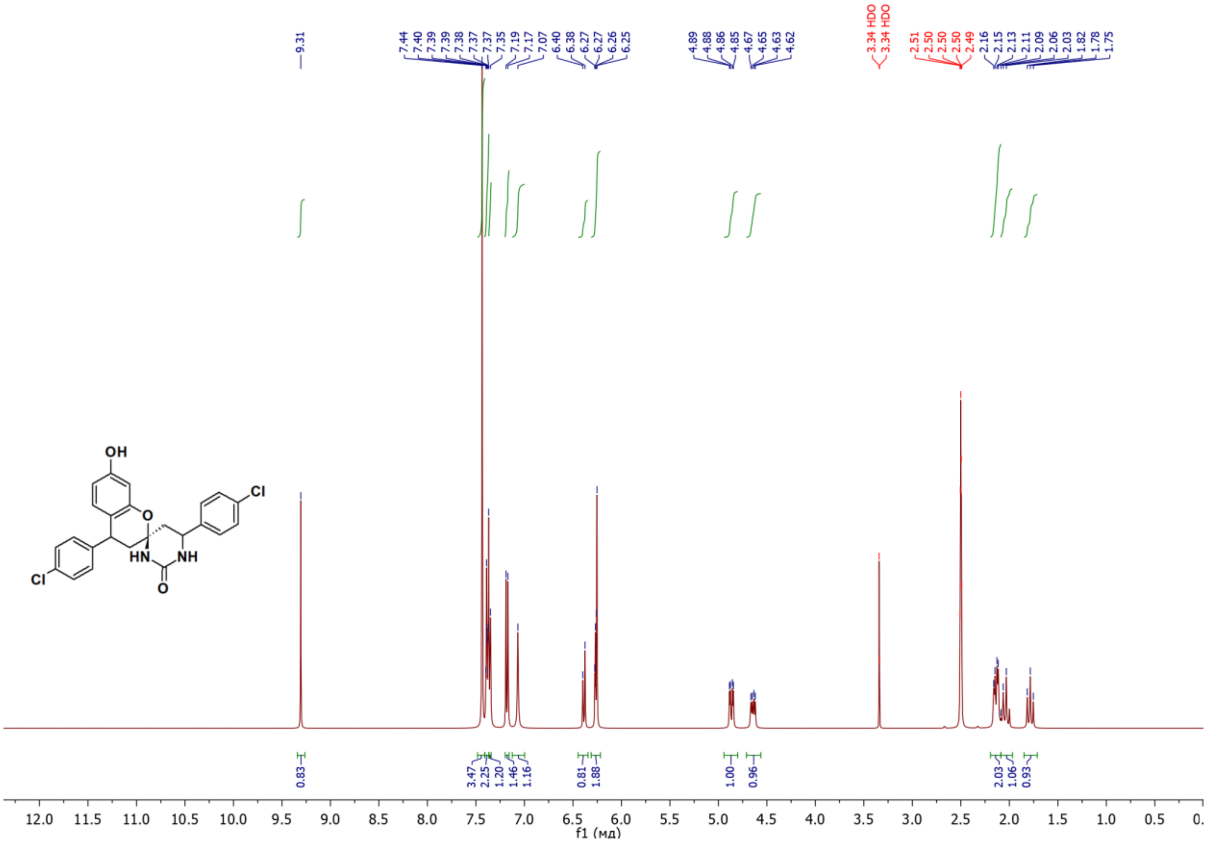

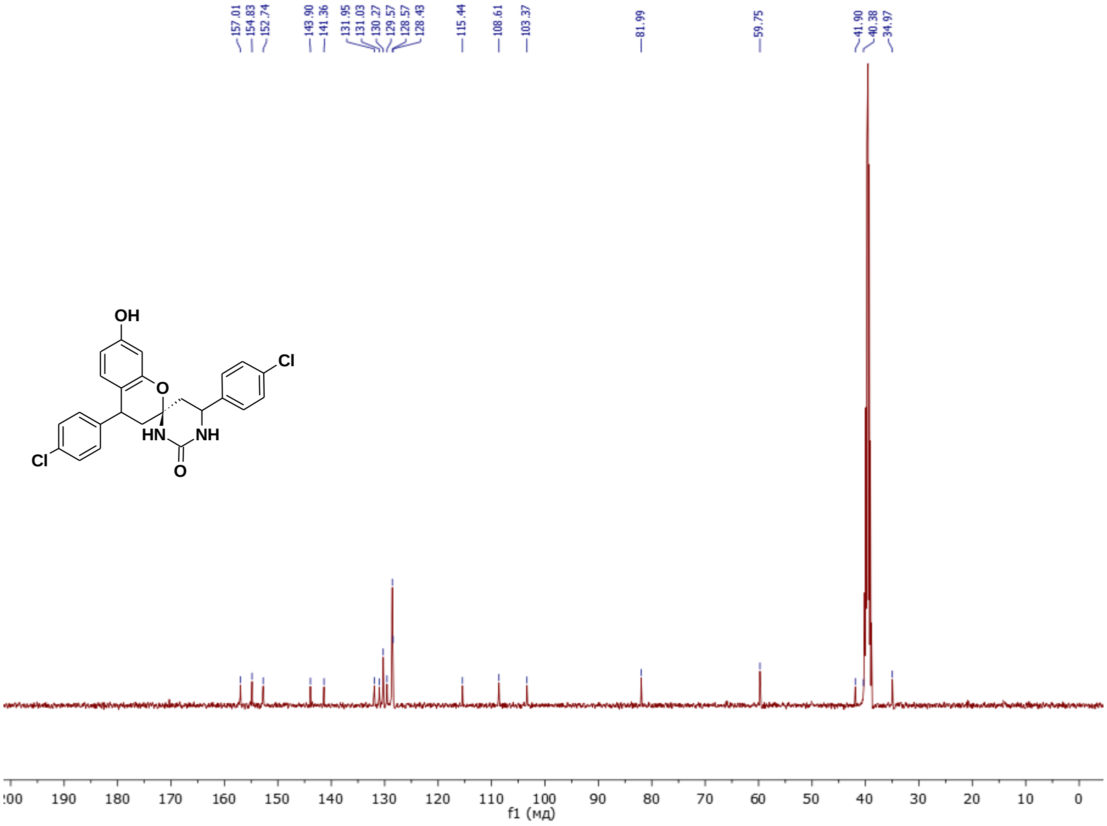

^{1}H and ^{13}C spectra of (2*S**,4*R**,6'*R**)-7,8-dihydroxy-4,6'-diphenyl-5',6'-dihydro-1'*H*-spiro[chromane-2,4'-pyrimidin]-2'(3'*H*)-one (**5g**)

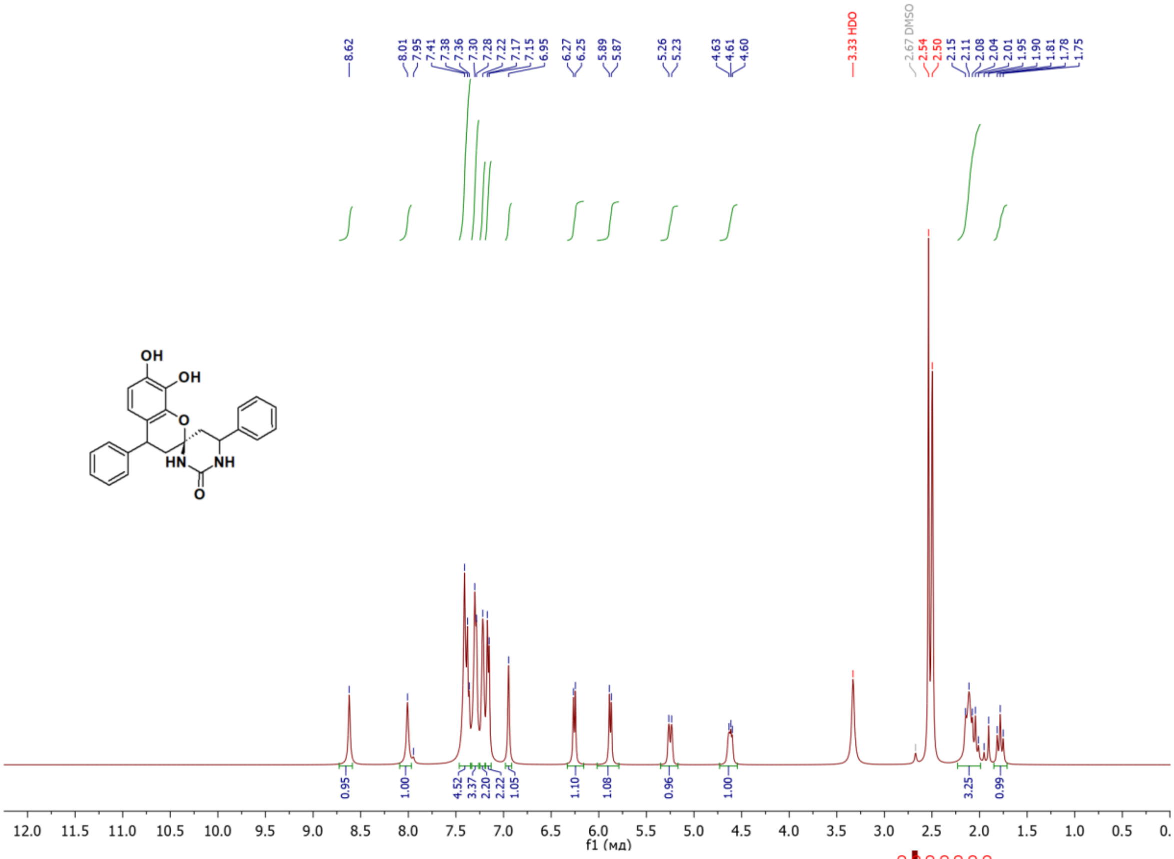

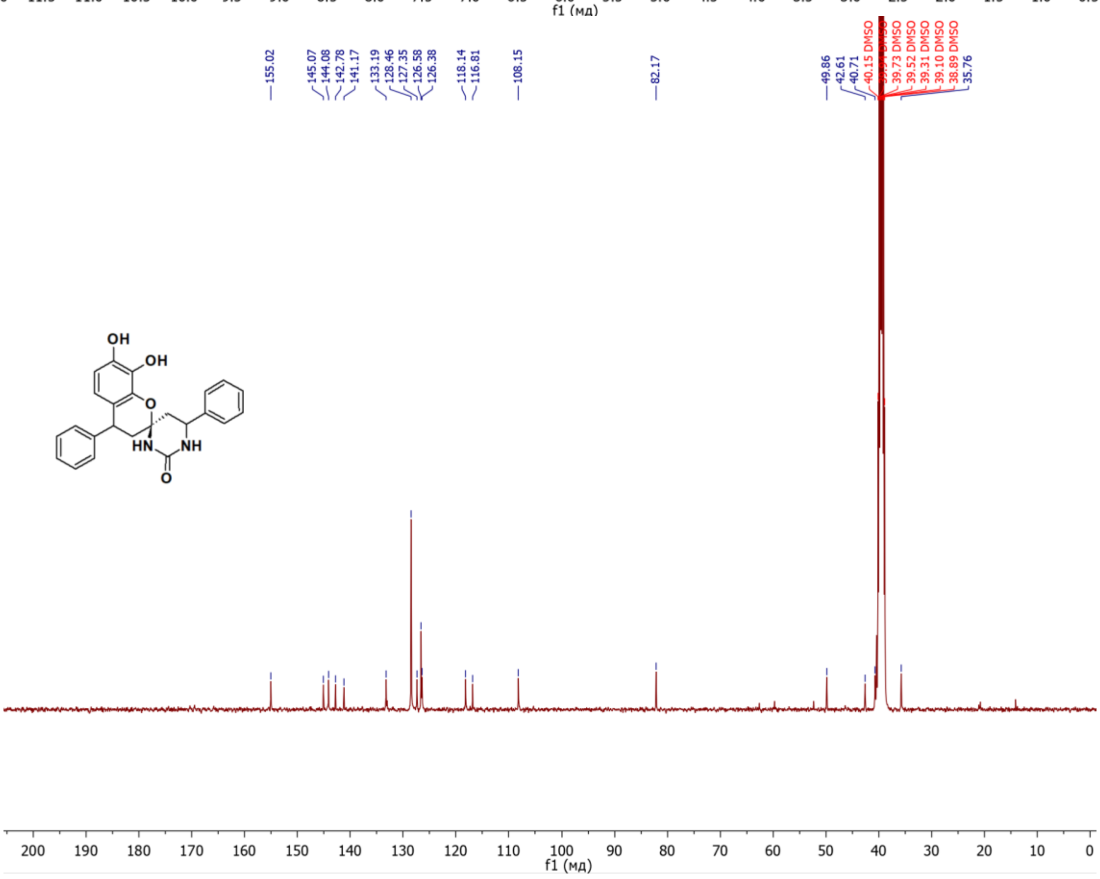

^{1}H and ^{13}C spectra of (2*R**,4*R**,6'*R**)-7,8-dihydroxy-4,6'-diphenyl-5',6'-dihydro-1'*H*-spiro[chromane-2,4'-pyrimidin]-2'(3'*H*)-one (**6g**)

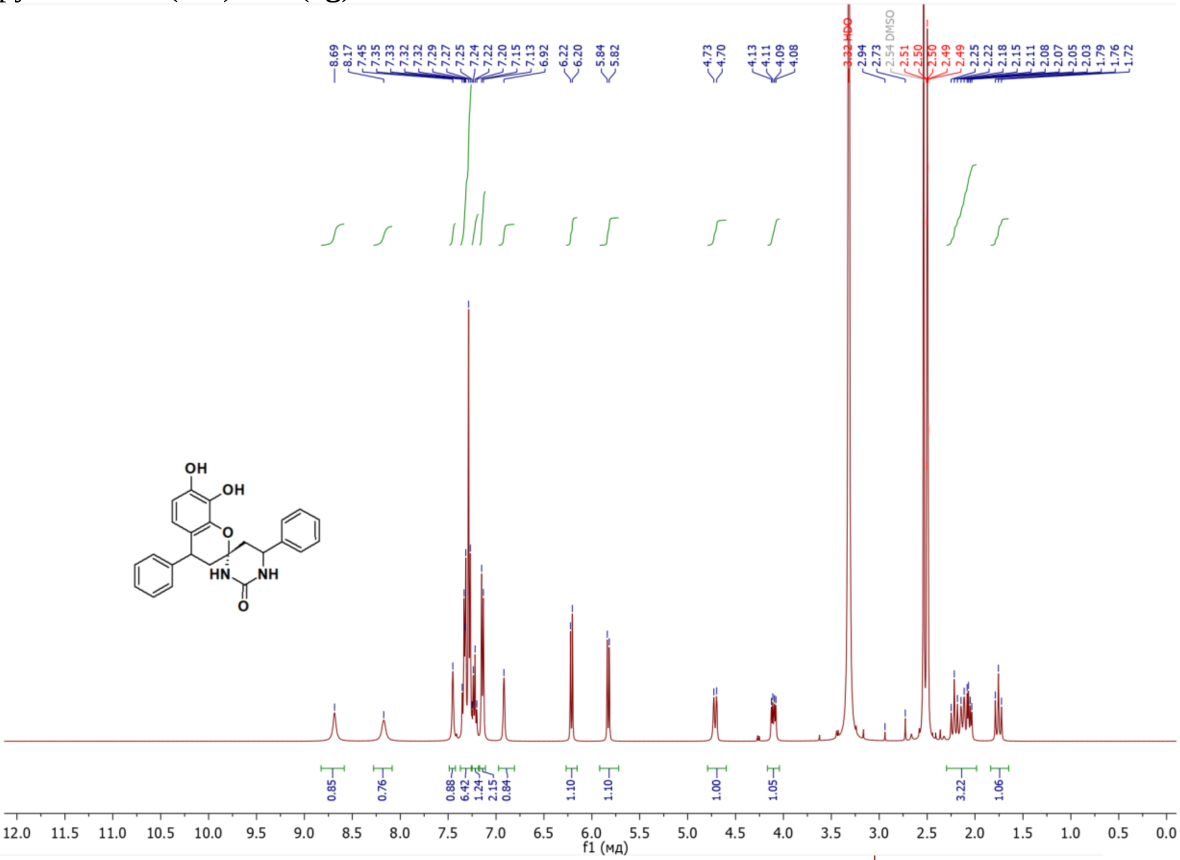

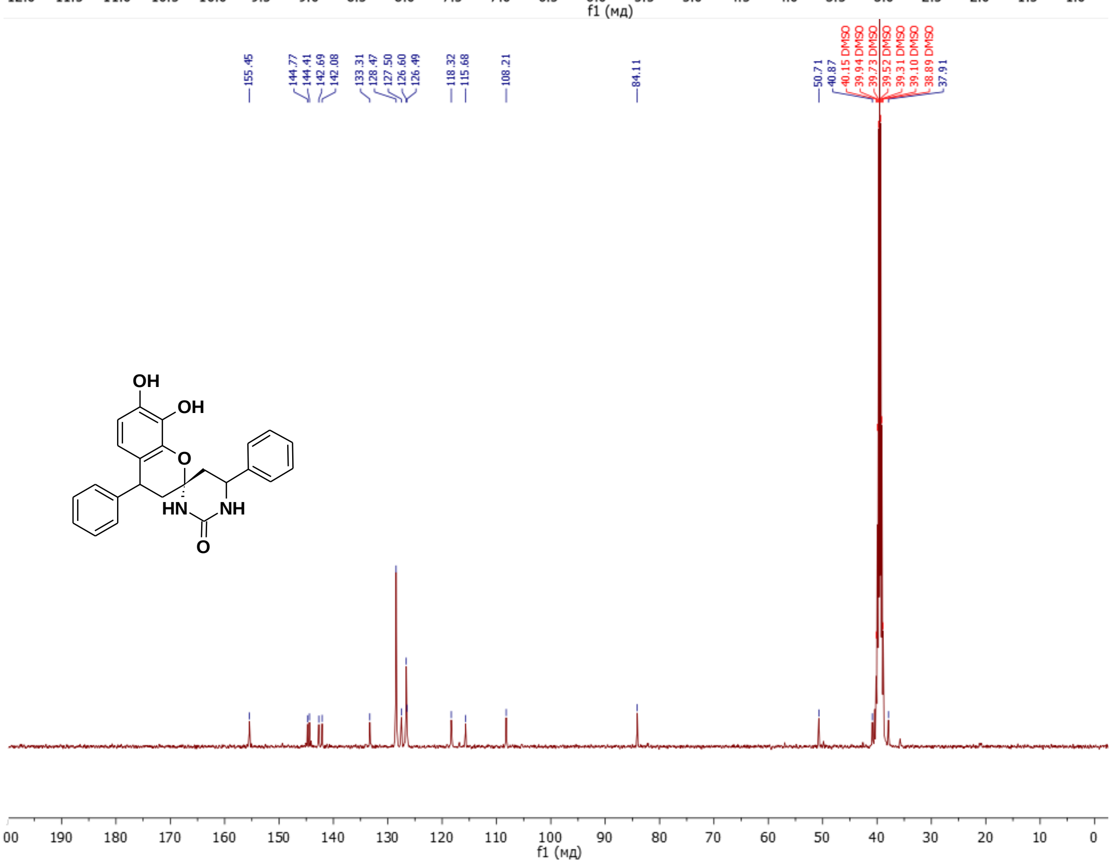

^{1}H and ^{13}C spectra of (2*S**,4*R**,6'*R**)-4-(4-chlorophenyl)-7,8-dihydroxy-6'-phenyl-5',6'-dihydro-1'*H*-spiro[chromane-2,4'-pyrimidin]-2'(3'*H*)-one (**5h**)

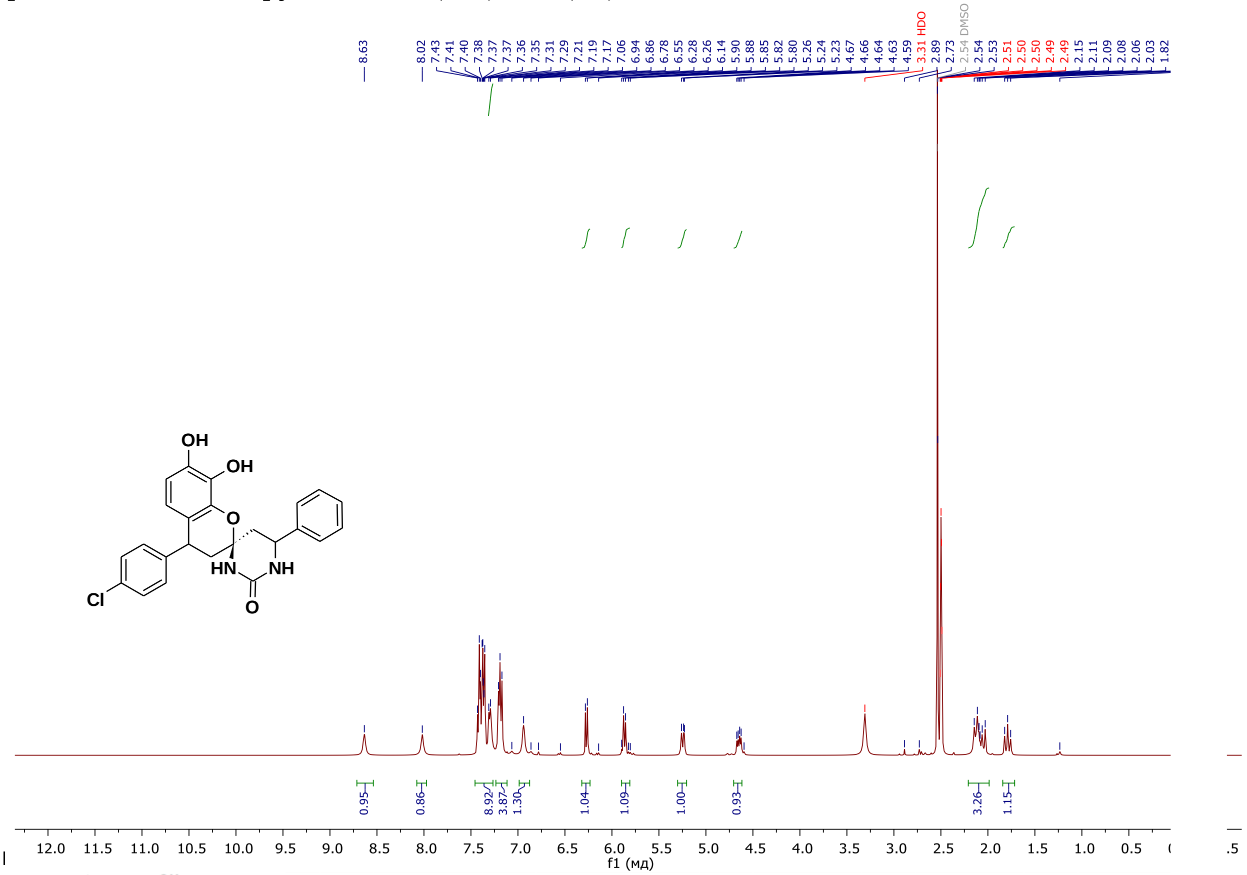

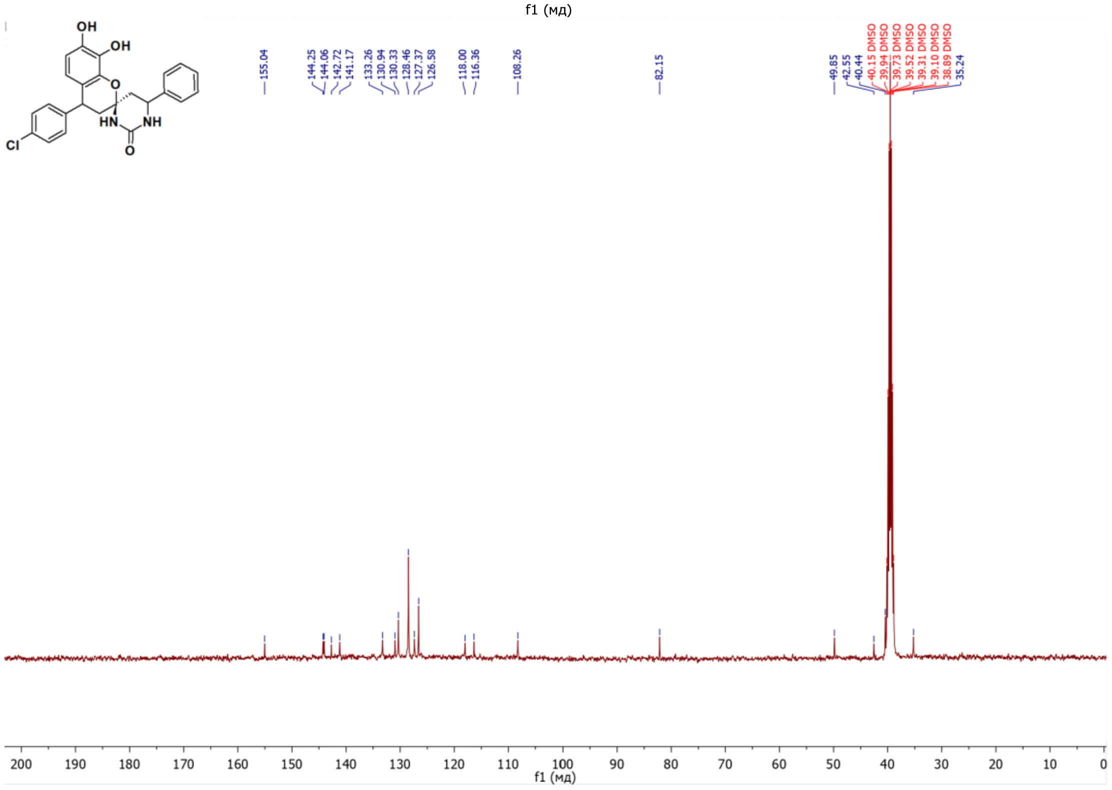

^{1}H and ^{13}C spectra of (2*R**,4*R**,6'*R**)-4-(4-chlorophenyl)-7,8-dihydroxy-6'-phenyl-5',6'-dihydro-1'*H*-spiro[chromane-2,4'-pyrimidin]-2'(3'*H*)-one (**6h**)

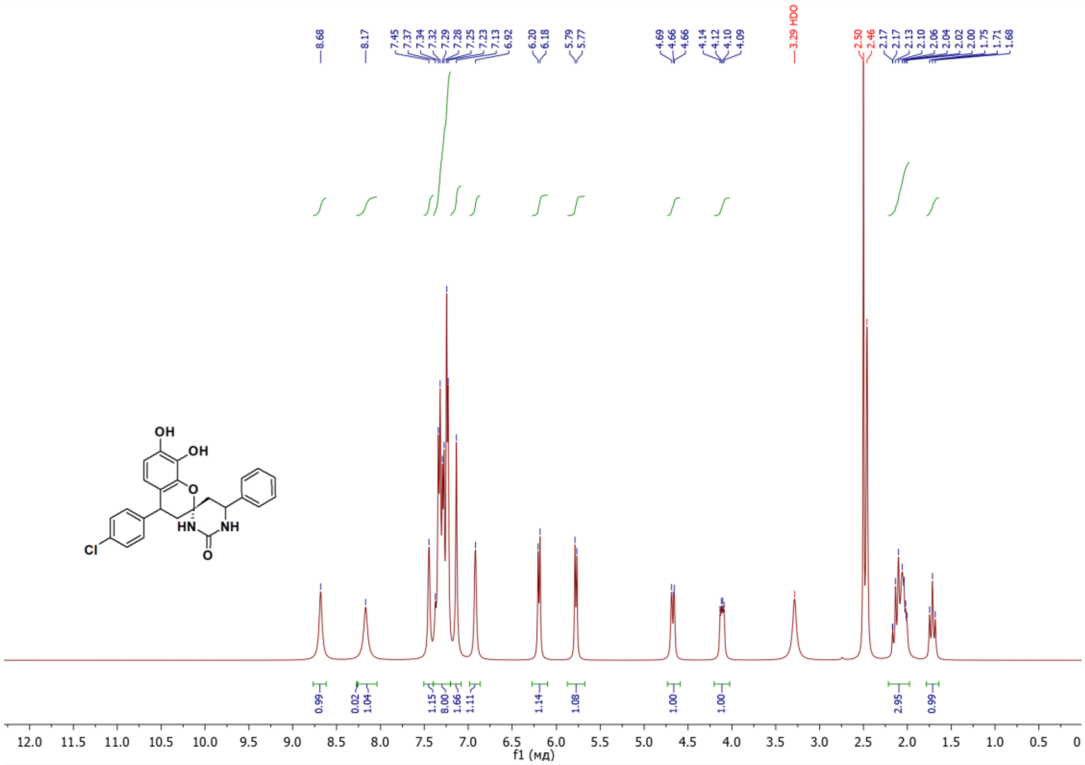

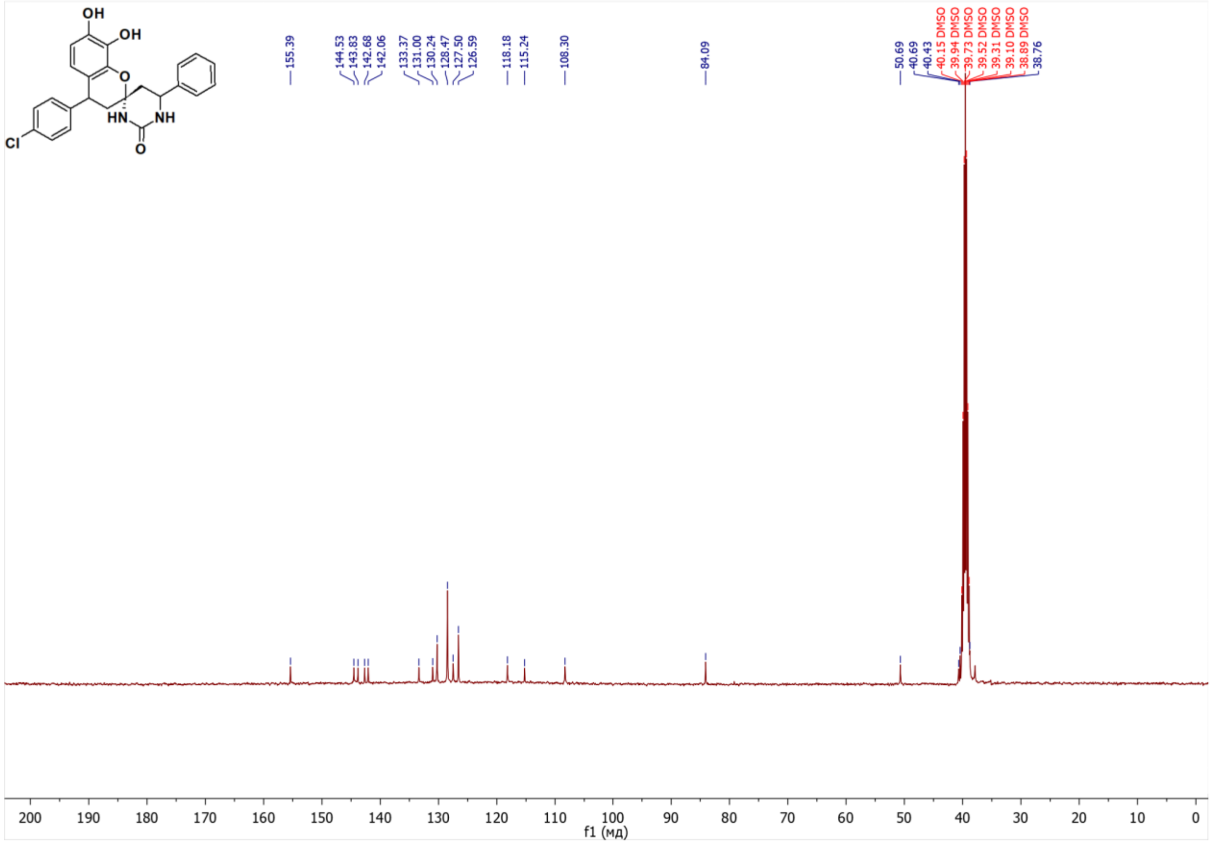

^{1}H, ^{13}C and NOESY spectra of (2*S**,4*R**,6'*R**)-7,8-dihydroxy-6'-phenyl-4-(*p*-tolyl)-5',6'-dihydro-1'*H*-spiro[chromane-2,4'-pyrimidin]-2'(3'*H*)-one (**5i)**

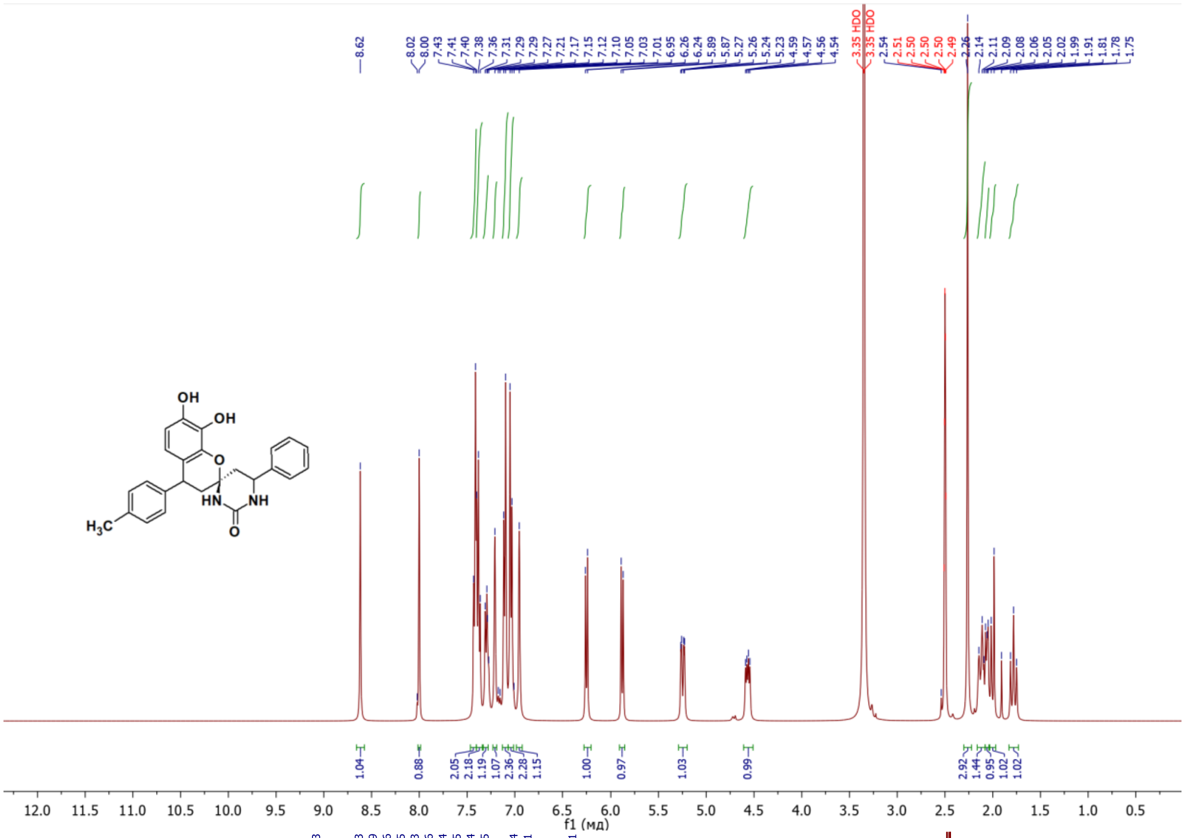

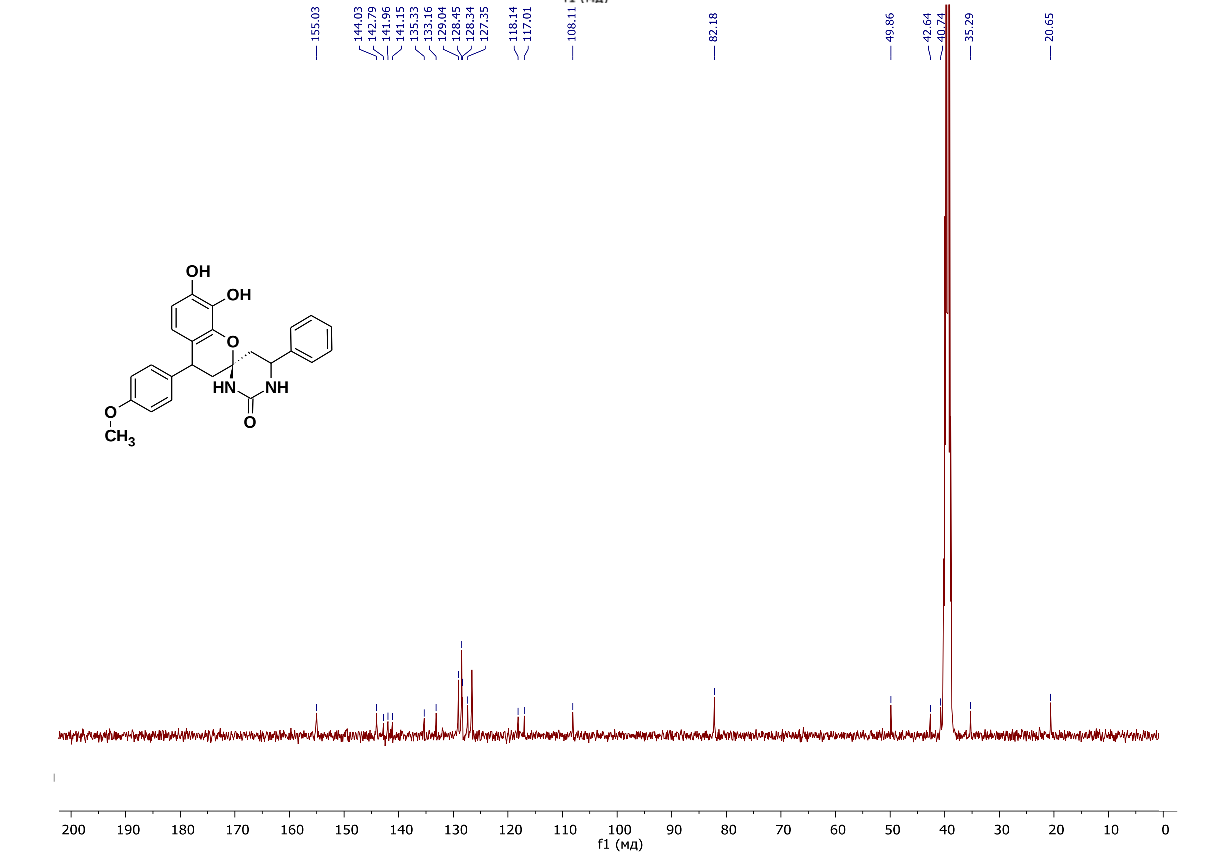

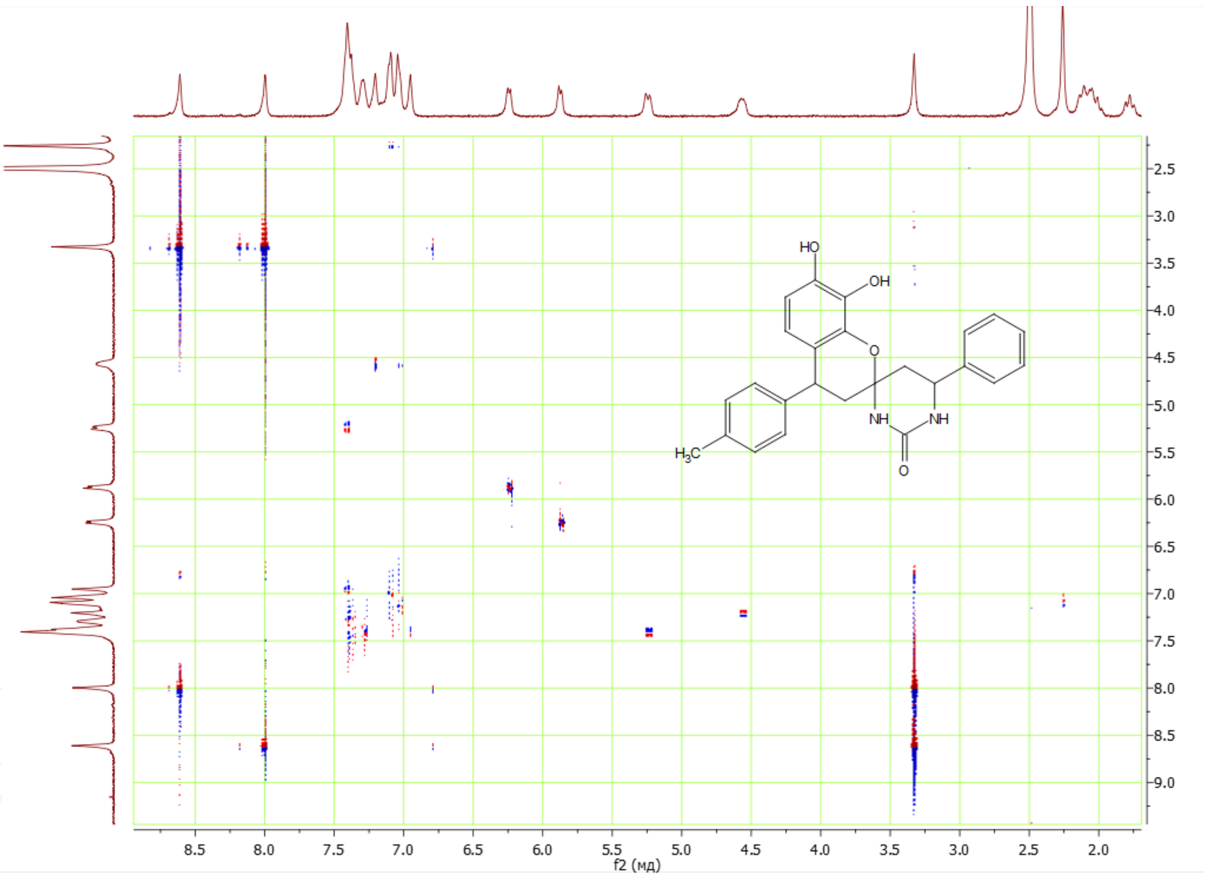

HO
OH
O
NH
NH
O
H3C
f2 (мд)

^{1}H and ^{13}C spectra of(2*S**,4*R**,6'*R**)-7,8-dihydroxy-4-(4-methoxyphenyl)-6'-phenyl-5',6'-dihydro-1'*H*-spiro[chromane-2,4'-pyrimidin]-2'(3'*H*)-one (**5j**)

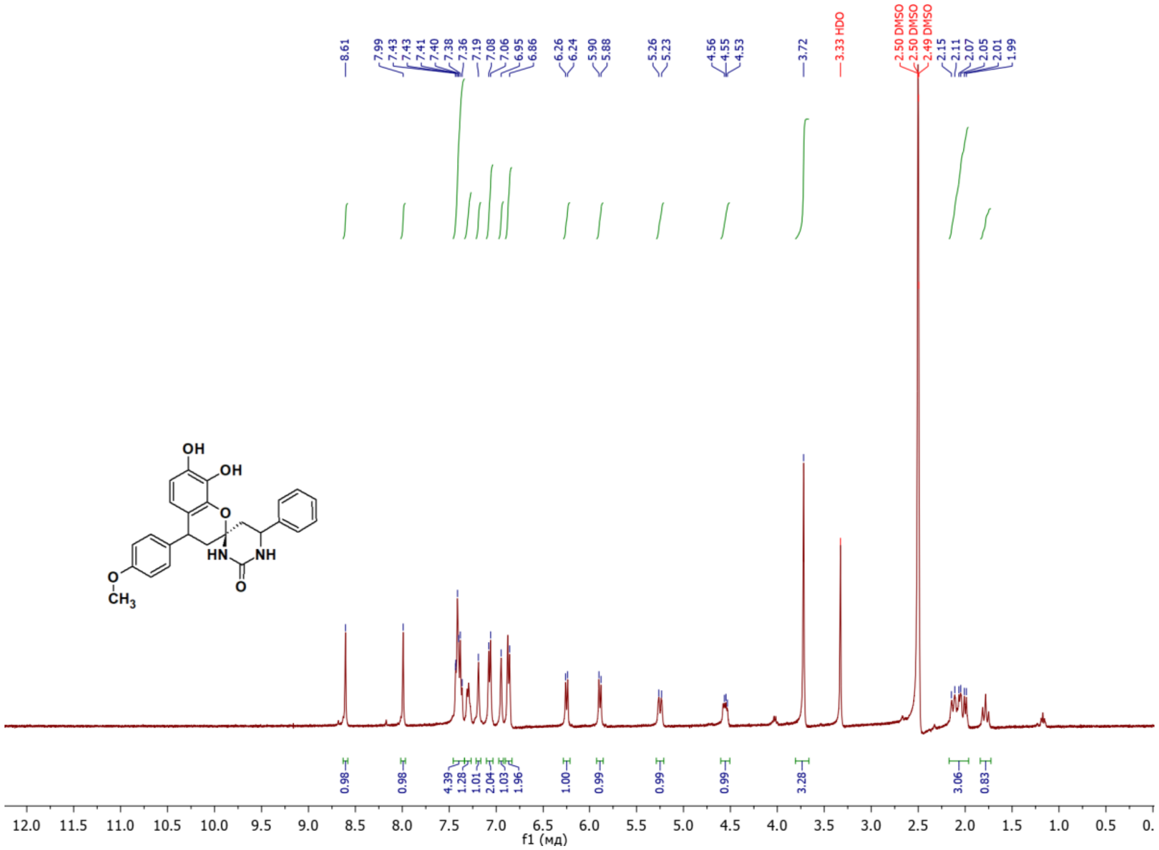

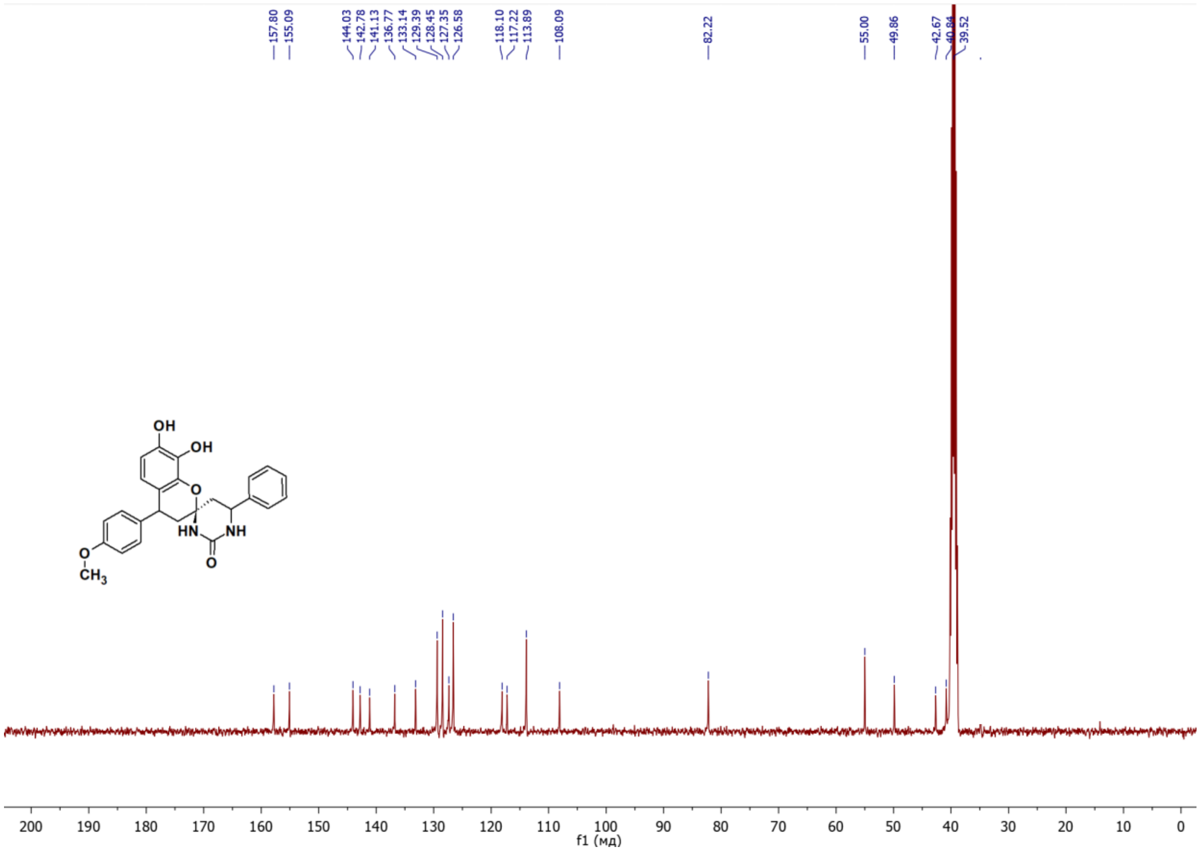

^{1}H,^{13}C and NOESYspectra of (2*S**,4*R**,6'*R**)-4,6'-bis(4-chlorophenyl)-7,8-dihydroxy-5',6'-dihydro-1'*H*-spiro[chromane-2,4'-pyrimidin]-2'(3'*H*)-one (**5k**)

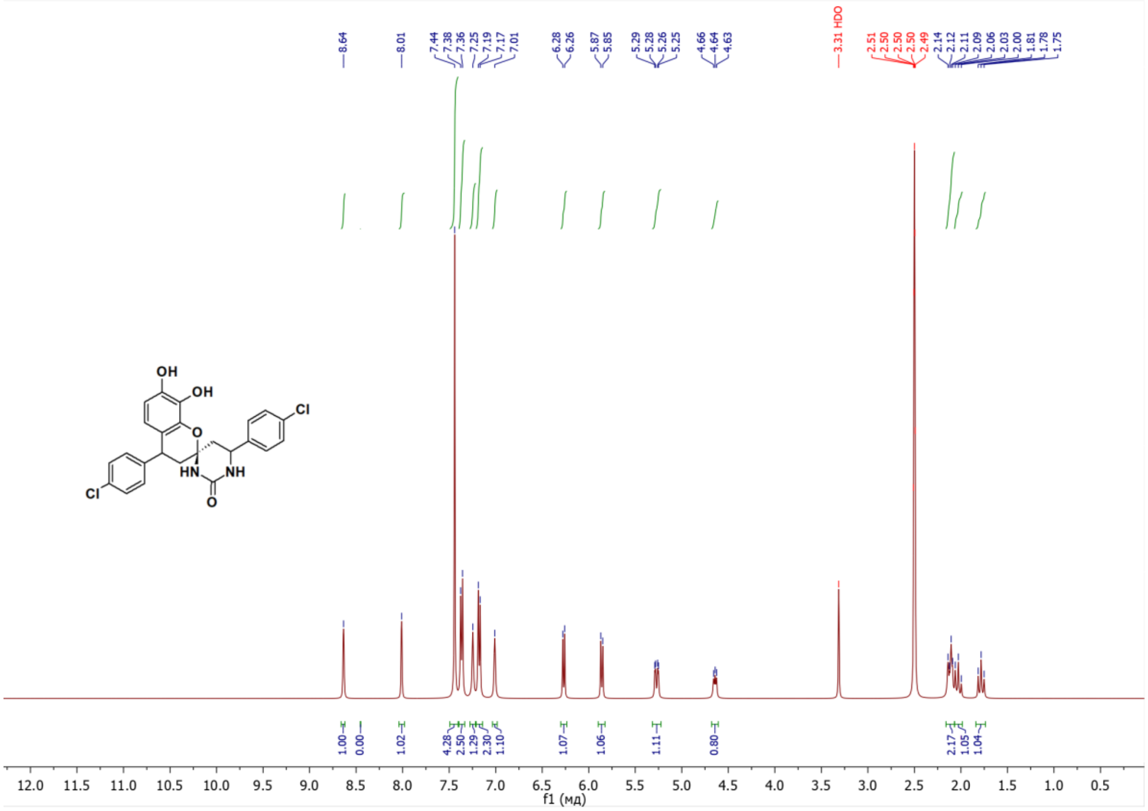

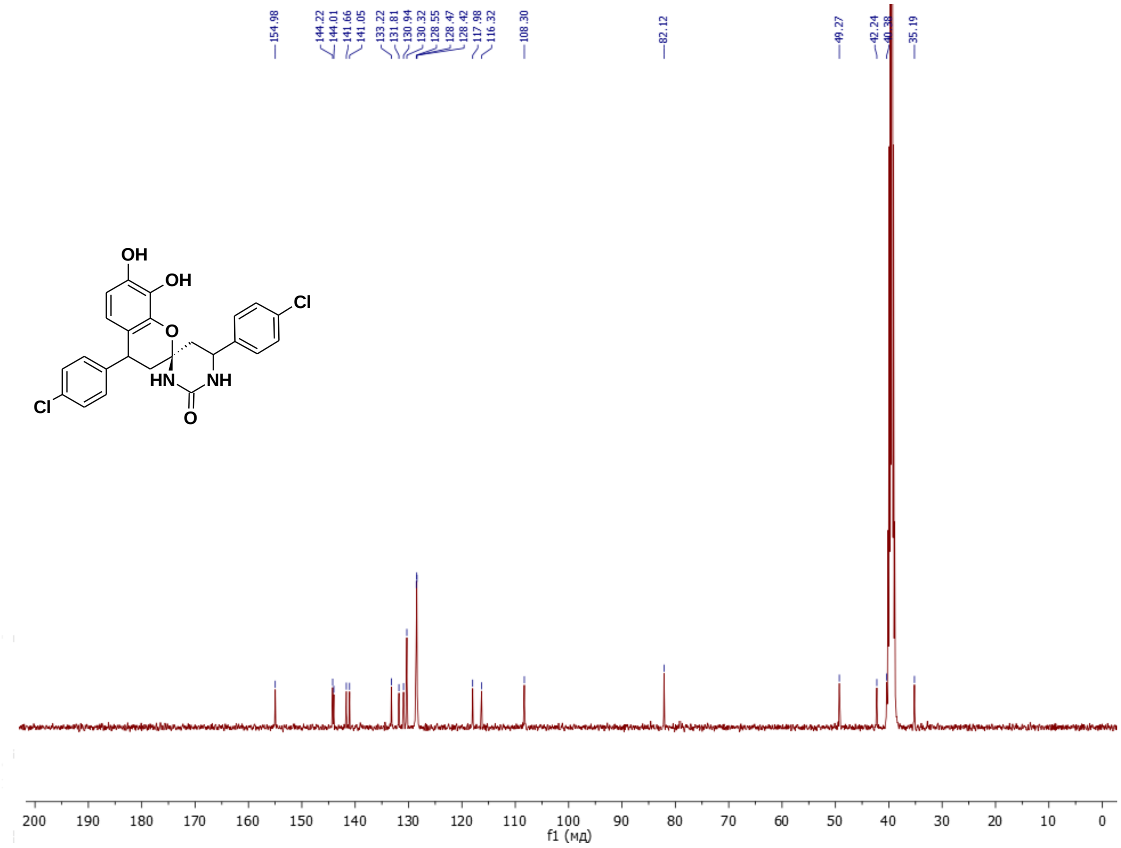

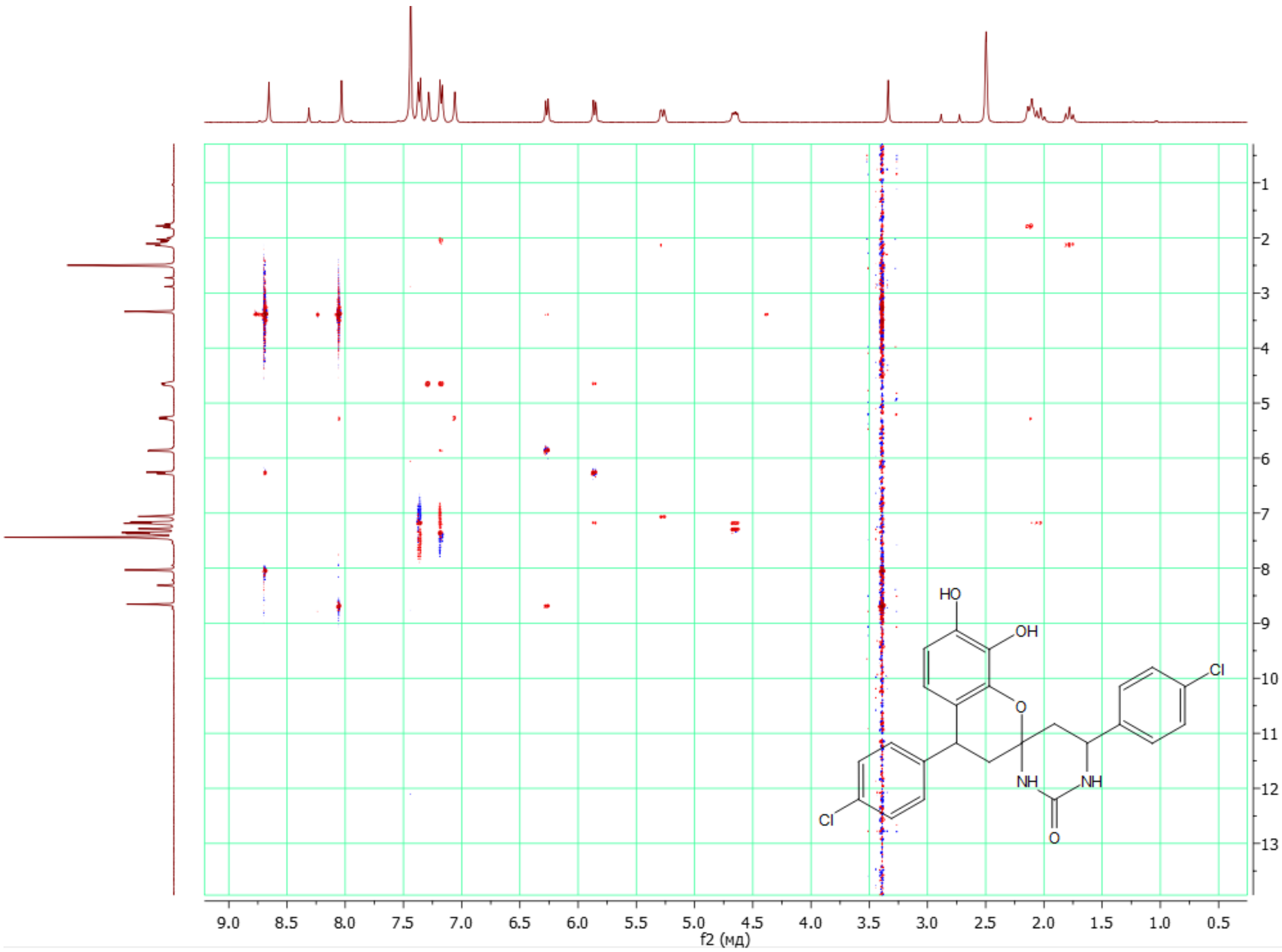
f2 (мд)
HO
OH
O
NH
NH
O
Cl
Cl

^{1}H,^{13}C and NOESY spectra of(2*R**,4*R**,6'*R**)-4,6'-bis(4-chlorophenyl)-7,8-dihydroxy-5',6'-dihydro-1'*H*-spiro[chromane-2,4'-pyrimidin]-2'(3'*H*)-one (**6k**)

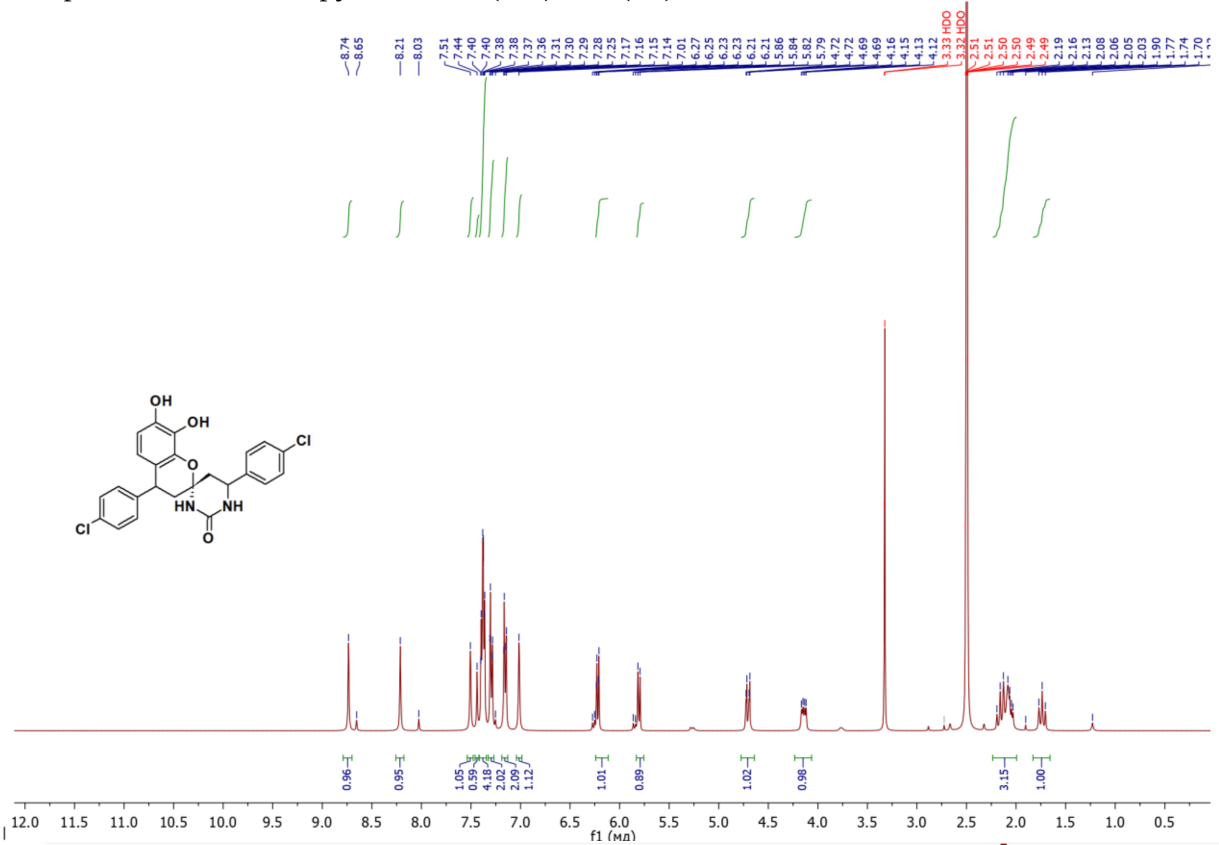

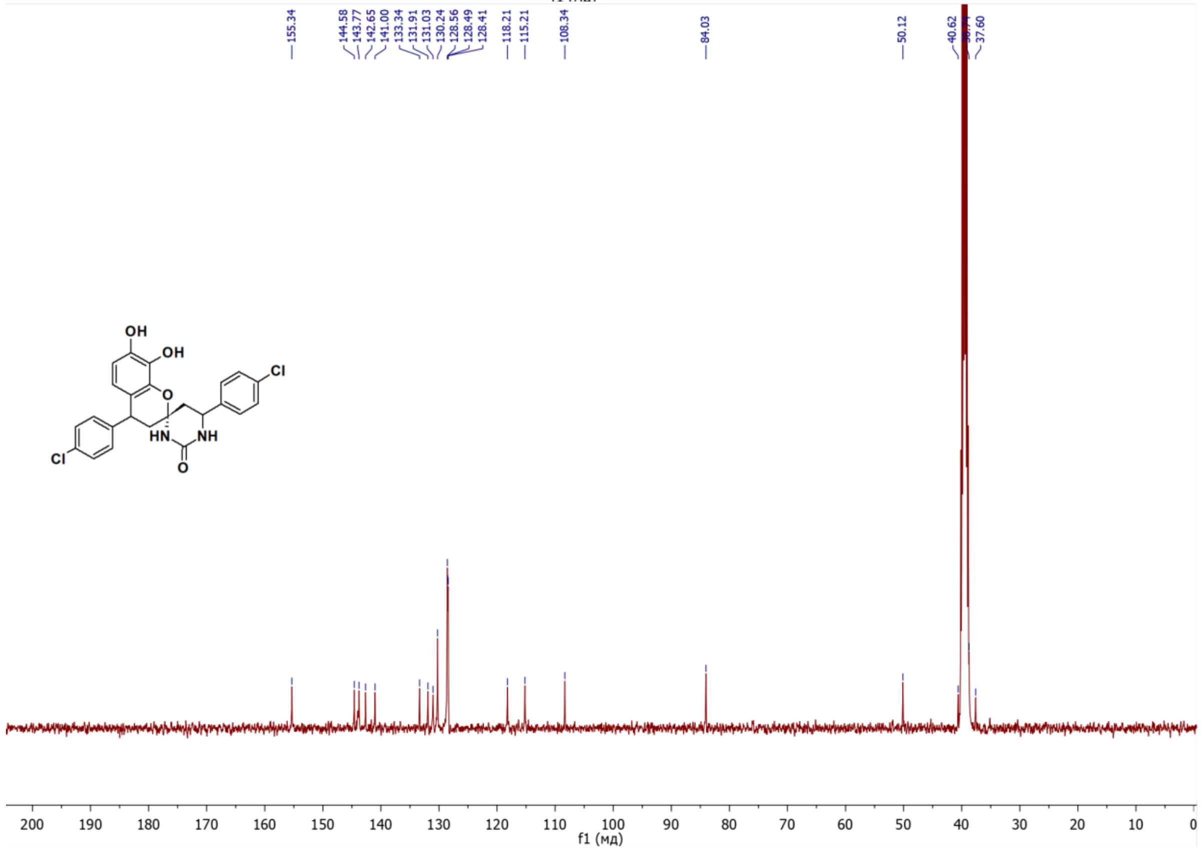

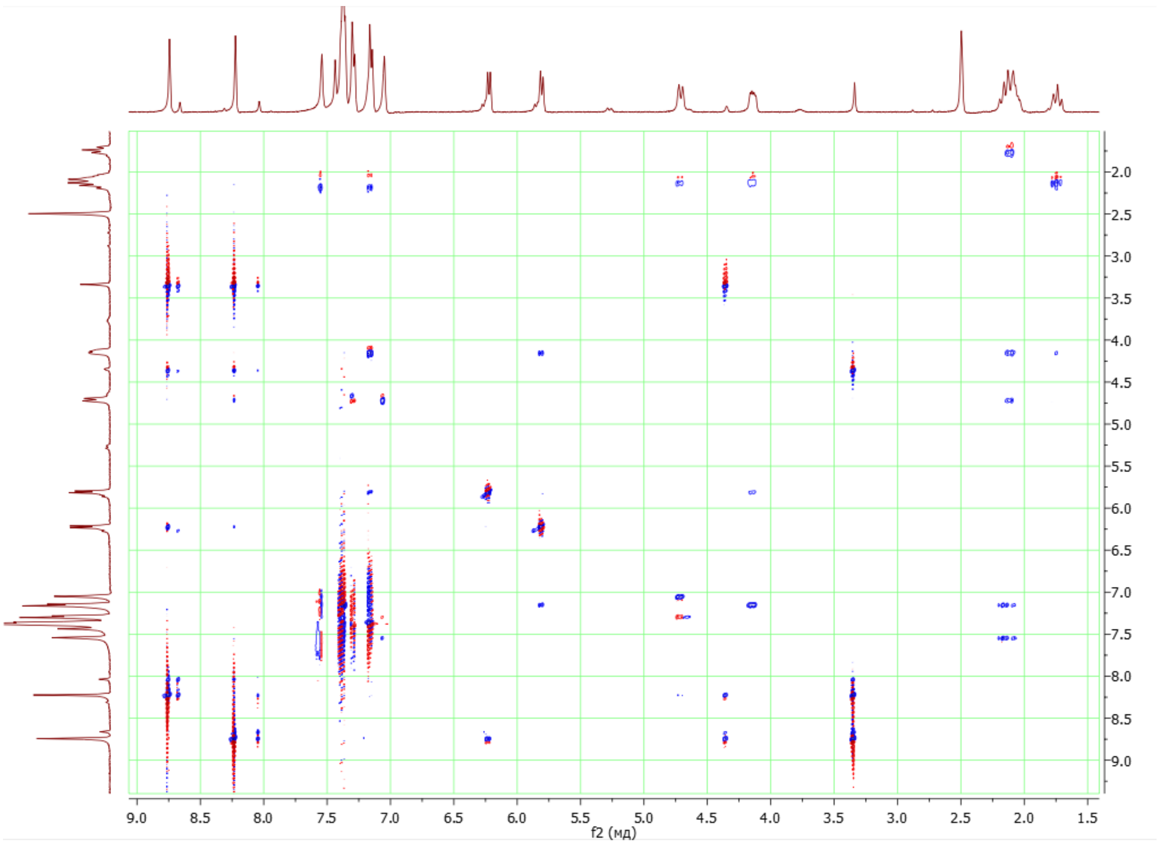

f2 (мд)
9.0
8.5
8.0
7.5
7.0
6.5
6.0
5.5
5.0
4.5
4.0
3.5
3.0
2.5
2.0
1.5

S3. Computational ADME data of spiro[chromane-2,4'-pyrimidin]-2'(3'*H*)-ones from SwissADME© server

(2*S**,4*R**,6'*R**)-4-(4-chlorophenyl)-7-hydroxy-6'-phenyl-5',6'-dihydro-1'*H*-spiro[chromane-2,4'-pyrimidin]-2'(3'*H*)-one (**5b**)

Molecule 1

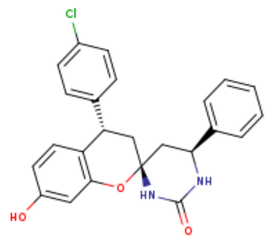

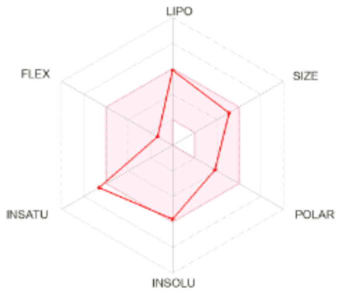

SMILES O=C1N[C@@H](C[C@]2(N1)Oc1cc(O)ccc1[C@@H](C2)c1ccc(cc1)Cl)c1ccccc1

Physicochemical Properties	
Formula	C24H21ClN2O3
Molecular weight	420.89 g/mol
Num. heavy atoms	30
Num. arom. heavy atoms	18
Fraction Csp3	0.21
Num. rotatable bonds	2
Num. H-bond acceptors	3
Num. H-bond donors	3
Molar Refractivity	122.97
TPSA	70.59 Å²

Lipophilicity	
Log $P_{o/w}$ (iLOGP)	2.63
Log $P_{o/w}$ (XLOGP3)	4.79
Log $P_{o/w}$ (WLOGP)	4.01
Log $P_{o/w}$ (MLOGP)	3.70
Log $P_{o/w}$ (SILICOS-IT)	4.02
Consensus Log $P_{o/w}$	3.83

Water Solubility	
Log *S* (ESOL)	-5.78
Solubility	7.00e-04 mg/ml ; 1.66e-06 mol/l
Class	Moderately soluble
Log *S* (Ali)	-6.00
Solubility	4.17e-04 mg/ml ; 9.90e-07 mol/l
Class	Poorly soluble
Log *S* (SILICOS-IT)	-8.30
Solubility	2.13e-06 mg/ml ; 5.06e-09 mol/l
Class	Poorly soluble

Pharmacokinetics	
GI absorption	High
BBB permeant	Yes
P-gp substrate	Yes
CYP1A2 inhibitor	No
CYP2C19 inhibitor	Yes
CYP2C9 inhibitor	Yes
CYP2D6 inhibitor	Yes
CYP3A4 inhibitor	Yes
Log K_p (skin permeation)	-5.47 cm/s

Druglikeness	
Lipinski	Yes; 0 violation
Ghose	Yes
Veber	Yes
Egan	Yes
Muegge	Yes
Bioavailability Score	0.55

Medicinal Chemistry	
PAINS	0 alert
Brenk	0 alert
Leadlikeness	No; 2 violations: MW>350, XLOGP3>3.5
Synthetic accessibility	4.66

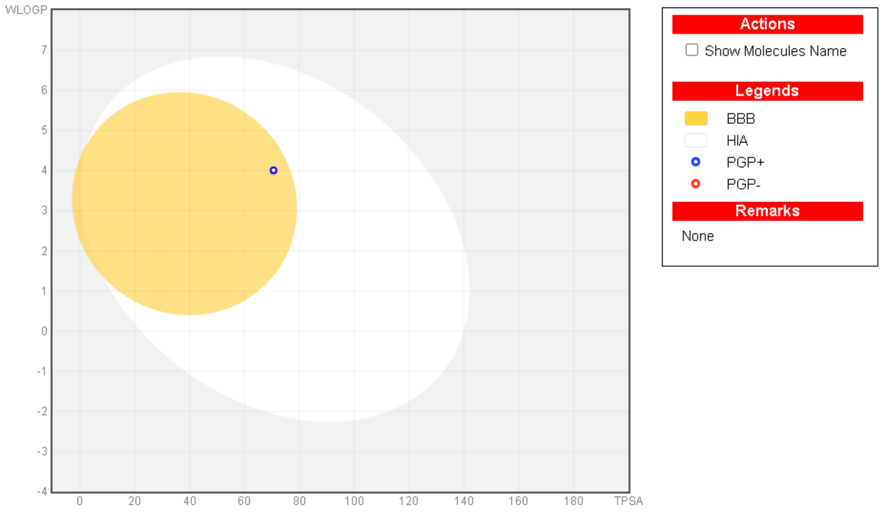

(2*S**,4*R**,6'*R**)-4,6'-bis(4-chlorophenyl)-7-hydroxy-5',6'-dihydro-1'*H*-spiro[chromane-2,4'-pyrimidin]-2'(3'*H*)-one (**5f**)

Molecule 1

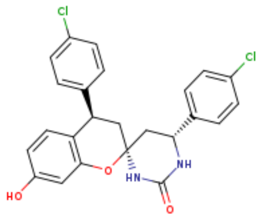

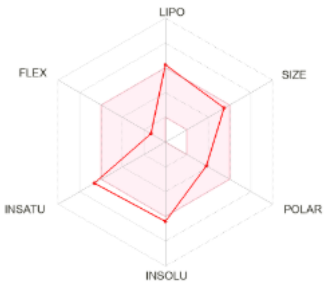

SMILES Clc1ccc(cc1)[C@@H]1NC(=O)N[C@]2(C1)Oc1cc(O)ccc1[C@H](C2)c1ccc(cc1)Cl

Physicochemical Properties	
Formula	C24H20Cl2N2O3
Molecular weight	455.33 g/mol
Num. heavy atoms	31
Num. arom. heavy atoms	18
Fraction Csp3	0.21
Num. rotatable bonds	2
Num. H-bond acceptors	3
Num. H-bond donors	3
Molar Refractivity	127.98
TPSA	70.59 Å²

Lipophilicity	
Log $P_{o/w}$ (iLOGP)	3.18
Log $P_{o/w}$ (XLOGP3)	5.42
Log $P_{o/w}$ (WLOGP)	4.67
Log $P_{o/w}$ (MLOGP)	4.17
Log $P_{o/w}$ (SILICOS-IT)	4.65
Consensus Log $P_{o/w}$	4.42

Water Solubility	
Log *S* (ESOL)	-6.38
Solubility	1.92e-04 mg/ml ; 4.21e-07 mol/l
Class	Poorly soluble
Log *S* (Ali)	-6.66
Solubility	1.00e-04 mg/ml ; 2.20e-07 mol/l
Class	Poorly soluble
Log *S* (SILICOS-IT)	-8.88
Solubility	6.03e-07 mg/ml ; 1.32e-09 mol/l
Class	Poorly soluble

Pharmacokinetics	
GI absorption	High
BBB permeant	Yes
P-gp substrate	Yes
CYP1A2 inhibitor	No
CYP2C19 inhibitor	Yes
CYP2C9 inhibitor	Yes
CYP2D6 inhibitor	Yes
CYP3A4 inhibitor	No
Log K_p (skin permeation)	-5.23 cm/s

Druglikeness	
Lipinski	Yes; 1 violation: MLOGP>4.15
Ghose	Yes
Veber	Yes
Egan	Yes
Muegge	No; 1 violation: XLOGP3>5
Bioavailability Score	0.55

Medicinal Chemistry	
PAINS	0 alert
Brenk	0 alert
Leadlikeness	No; 2 violations: MW>350, XLOGP3>3.5
Synthetic accessibility	4.67

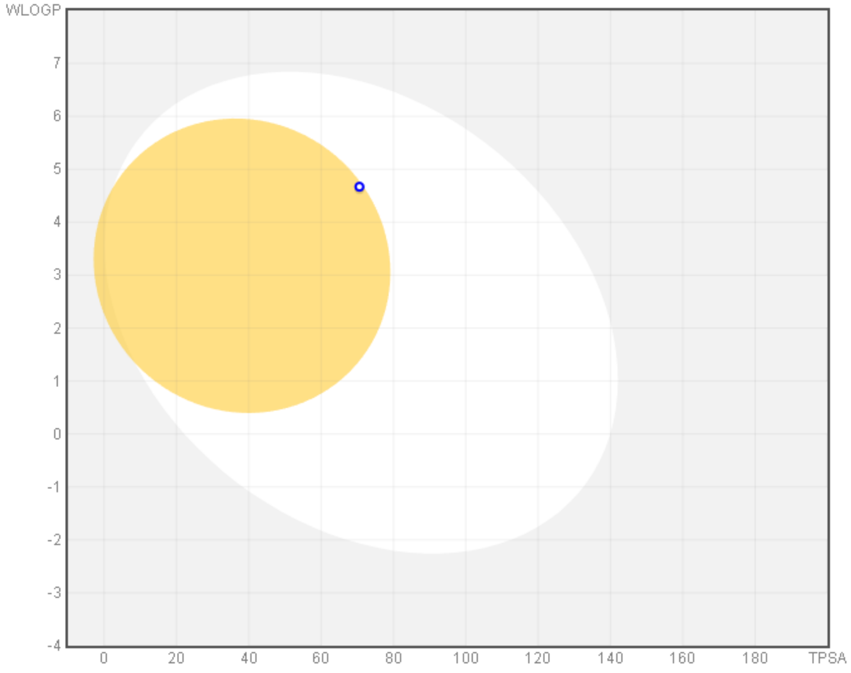

Actions

☐ Show Molecules Name

Legends

BBB

HIA

PGP+

PGP-

Remarks

None

(2S*,4*R**,6'*R**)-4-(4-chlorophenyl)-7,8-dihydroxy-6'-phenyl-5'',6'-dihydro-1'*H*-spiro[chromane-2,4'-pyrimidin]-2'(3'*H*)-one **(5h)**

Molecule 1

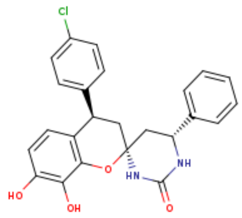

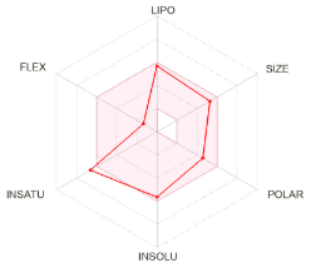

SMILES O=C1N[C@H](C[C@]2(N1)C[C@H](c1ccc(cc1)Cl)c1c(O2)c(O)c(cc1)O)c1ccccc1

Physicochemical Properties	
Formula	C24H21ClN2O4
Molecular weight	436.89 g/mol
Num. heavy atoms	31
Num. arom. heavy atoms	18
Fraction Csp3	0.21
Num. rotatable bonds	2
Num. H-bond acceptors	4
Num. H-bond donors	4
Molar Refractivity	124.99
TPSA	90.82 Å²

Lipophilicity	
Log $P_{o/w}$ (iLOGP)	2.82
Log $P_{o/w}$ (XLOGP3)	4.44
Log $P_{o/w}$ (WLOGP)	3.72
Log $P_{o/w}$ (MLOGP)	3.15
Log $P_{o/w}$ (SILICOS-IT)	3.53
Consensus Log $P_{o/w}$	3.53

Water Solubility	
Log *S* (ESOL)	-5.64
Solubility	9.93e-04 mg/ml ; 2.27e-06 mol/l
Class	Moderately soluble
Log *S* (Ali)	-6.07
Solubility	3.75e-04 mg/ml ; 8.59e-07 mol/l
Class	Poorly soluble
Log *S* (SILICOS-IT)	-7.71
Solubility	8.60e-06 mg/ml ; 1.97e-08 mol/l
Class	Poorly soluble

Pharmacokinetics	
GI absorption	High
BBB permeant	No
P-gp substrate	Yes
CYP1A2 inhibitor	No
CYP2C19 inhibitor	Yes
CYP2C9 inhibitor	Yes
CYP2D6 inhibitor	No
CYP3A4 inhibitor	No
Log K_p (skin permeation)	-5.81 cm/s

Druglikeness	
Lipinski	Yes; 0 violation
Ghose	Yes
Veber	Yes
Egan	Yes
Muegge	Yes
Bioavailability Score	0.55

Medicinal Chemistry	
PAINS	1 alert: catechol_A
Brenk	1 alert: catechol
Leadlikeness	No; 2 violations: MW>350, XLOGP3>3.5
Synthetic accessibility	4.76

Hide BOILED-Egg

Retrieve data: Powered by Chemaxon

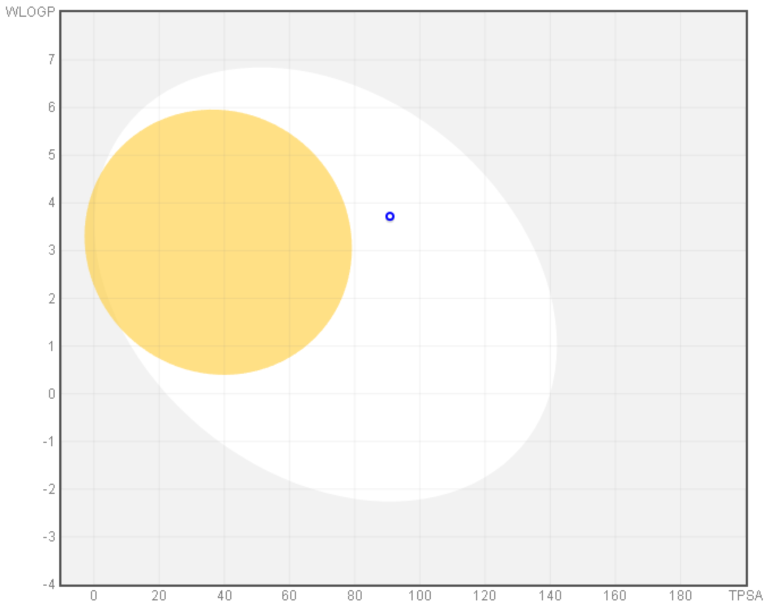

Actions

Show Molecules Name

Legends

BBB

HIA

PGP+

PGP-

Remarks

None

2*R**,4*R**,6'*R**)-4-(4-chlorophenyl)-7,8-dihydroxy-6'-phenyl-5',6'-dihydro-1'*H*-spiro[chromane-2,4'-pyrimidin]-2'(3'*H*)-one (**6h**)

Molecule 1

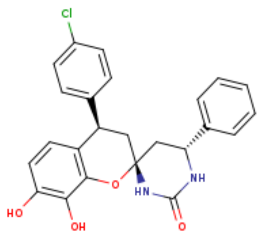

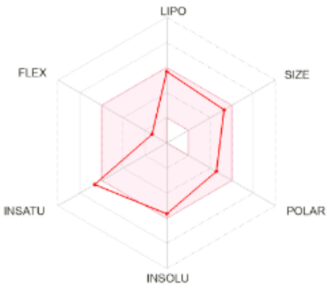

SMILES	O=C1N[C@H](C[C@@]2(N1)C[C@H](c1ccc(cc1)Cl)c1c(O2)c(O)c(cc1)O)c1ccccc1
**Physicochemical Properties**	
Formula	C24H21ClN2O4
Molecular weight	436.89 g/mol
Num. heavy atoms	31
Num. arom. heavy atoms	18
Fraction Csp3	0.21
Num. rotatable bonds	2
Num. H-bond acceptors	4
Num. H-bond donors	4
Molar Refractivity	124.99
TPSA	90.82 Å²
**Lipophilicity**	
Log $P_{o/w}$ (iLOGP)	2.50
Log $P_{o/w}$ (XLOGP3)	4.44
Log $P_{o/w}$ (WLOGP)	3.72
Log $P_{o/w}$ (MLOGP)	3.15
Log $P_{o/w}$ (SILICOS-IT)	3.53
Consensus Log $P_{o/w}$	3.47

**Water Solubility**	
Log *S* (ESOL)	-5.64
Solubility	9.93e-04 mg/ml ; 2.27e-06 mol/l
Class	Moderately soluble
Log *S* (Ali)	-6.07
Solubility	3.75e-04 mg/ml ; 8.59e-07 mol/l
Class	Poorly soluble
Log *S* (SILICOS-IT)	-7.71
Solubility	8.60e-06 mg/ml ; 1.97e-08 mol/l
Class	Poorly soluble
**Pharmacokinetics**	
GI absorption	High
BBB permeant	No
P-gp substrate	Yes
CYP1A2 inhibitor	No
CYP2C19 inhibitor	Yes
CYP2C9 inhibitor	Yes
CYP2D6 inhibitor	No
CYP3A4 inhibitor	Yes
Log K_p (skin permeation)	-5.81 cm/s
**Druglikeness**	
Lipinski	Yes; 0 violation
Ghose	Yes
Veber	Yes
Egan	Yes
Muegge	Yes
Bioavailability Score	0.55
**Medicinal Chemistry**	
PAINS	1 alert: catechol_A
Brenk	1 alert: catechol
Leadlikeness	No; 2 violations: MW>350, XLOGP3>3.5
Synthetic accessibility	4.76

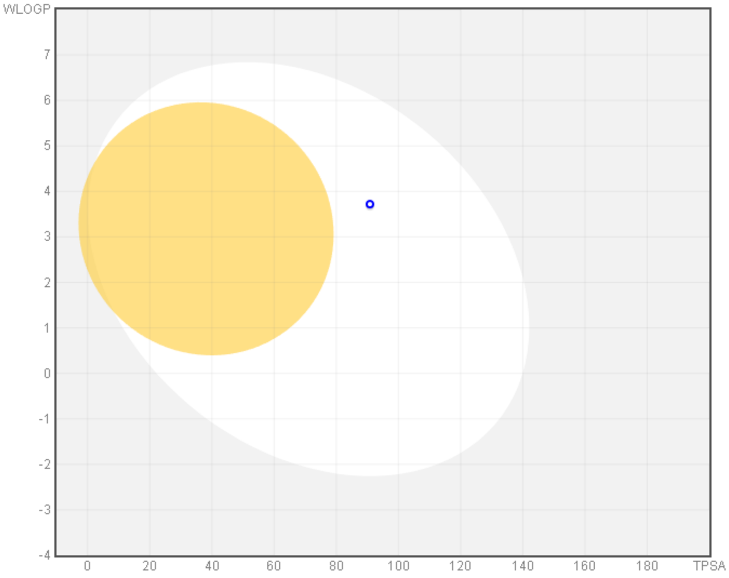

Actions

☐ Show Molecules Name

Legends

BBB

HIA

PGP+

PGP-

Remarks

None
